# DrpB (YedR) Is a Nonessential Cell Division Protein in Escherichia coli

**DOI:** 10.1128/JB.00284-20

**Published:** 2020-11-04

**Authors:** Atsushi Yahashiri, Jill T. Babor, Ariel L. Anwar, Ryan P. Bezy, Evan W. Piette, S. J. Ryan Arends, Ute Müh, Monica R. Steffen, Jeremy M. Cline, David N. Stanek, Steven D. Lister, Shauna M. Swanson, David S. Weiss

**Affiliations:** a Department of Microbiology and Immunology, University of Iowa Carver College of Medicine, Iowa City, Iowa, USA; b Department of Natural and Applied Sciences, Mount Mercy University, Cedar Rapids, Iowa, USA; University of Montreal

**Keywords:** Y genes, cytokinesis, *dedD*, divisome, *ftsEX*, septal ring, suppressor mutant

## Abstract

We report that the small Escherichia coli membrane protein DrpB (formerly YedR) is involved in cell division. We discovered DrpB in a screen for multicopy suppressors of a Δ*ftsEX* mutation that prevents divisome assembly when cells are plated on low ionic strength medium, such as lysogeny broth without NaCl. Characterization of DrpB revealed that (i) translation initiates at an ATG annotated as codon 22 rather than the GTG annotated as codon 1, (ii) DrpB localizes to the septal ring when cells are grown in medium of low ionic strength but localization is greatly reduced in medium of high ionic strength, (iii) overproduction of DrpB in a Δ*ftsEX* mutant background improves recruitment of the septal peptidoglycan synthase FtsI, implying multicopy suppression works by rescuing septal ring assembly, (iv) a Δ*drpB* mutant divides quite normally, but a Δ*drpB* Δ*dedD* double mutant has a strong division and viability defect, albeit only in medium of high ionic strength, and (v) DrpB homologs are found in E. coli and a few closely related enteric bacteria, but not outside this group. In sum, DrpB is a poorly conserved nonessential division protein that improves the efficiency of cytokinesis under suboptimal conditions. Proteins like DrpB are likely to be a widespread feature of the bacterial cell division apparatus, but they are easily overlooked because mutants lack obvious shape defects.

**IMPORTANCE** A thorough understanding of bacterial cell division requires identifying and characterizing all of the proteins that participate in this process. Our discovery of DrpB brings us one step closer to this goal in E. coli.

## Abstract (precis)

A thorough understanding of bacterial cell division requires identifying and characterizing all of the proteins that participate in this process. Our discovery of DrpB brings us one step closer to this goal in E. coli.

## INTRODUCTION

Cell division in Escherichia coli is carried out by the septal ring or divisome, a loosely defined assemblage of over 30 distinct proteins that form a contractile, ring-like structure at the midcell (1–3). About a dozen of these proteins are essential and have readily identifiable homologs in a wide range of bacteria; these proteins are considered to constitute the core of the division apparatus. Nevertheless, the core proteins are not sufficient for cytokinesis. Rather, they work together with multiple accessory proteins that play less critical and/or partially redundant roles. The importance of the nonessential division ring proteins, at least collectively, is underscored by the fact that they now outnumber their essential counterparts by over two to one, and new nonessential division ring proteins continue to be discovered (4–6).

The essential E. coli division proteins are mostly named Fts, for *f*ilamentation *t*emperature *s*ensitive. Most were discovered decades ago in screens for temperature-sensitive mutants that became filamentous and ultimately died under nonpermissive conditions (7, 8). In contrast, the nonessential division proteins have been discovered more recently and by a wider variety of approaches: bioinformatic searches for homology to known division proteins, screens for proteins that interact with known division proteins, screens for proteins that localize to the division site, and genetic screens for synthetic lethality or suppression in various *fts* mutant backgrounds (5, 6, 9–18).

Here, we report the discovery and initial characterization of a new nonessential E. coli division protein that we have named DrpB, for *d*ivision *r*ing *p*rotein *B*. (Note that the name DrpA was used 30 years ago for a protein thought at the time to be involved in DNA replication but now known to be a tRNA synthetase and designated ProS [19, 20].) We identified DrpB in a selection for multicopy suppressors of a Δ*ftsEX* null mutant. FtsEX is a pseudo ABC transporter that coordinates synthesis and processing of septal peptidoglycan (PG) (21–23). Importantly for the present study, FtsEX is also a critical septal ring assembly factor (24). In the absence of FtsEX, E. coli assembles an incomplete septal ring that contains so-called “early” recruits like FtsZ and FtsA but lacks the “late” recruits that are more directly involved in synthesis and processing of septal peptidoglycan, such as the transpeptidase FtsI. The severity of the localization and viability defects depend on growth conditions (24). Thus, an E. coli Δ*ftsEX* mutant can form colonies when plated on lysogeny broth (LB) containing the usual 5 to 10 g of NaCl per liter, although microscopy reveals many of the cells in these colonies display filamentation and chaining defects. However, an *ftsEX* null mutant is not viable when plated on LB made without NaCl (here termed LB0N for LB “zero” NaCl). Colony formation on LB0N can be rescued in a variety of ways. One is by raising the osmotic strength of the medium with sucrose or proline (25). Alternatively, viability can be improved by lowering the temperature or by overproducing various division proteins, including FtsQAZ, FtsN, FtsP or DapE (16, 25). In addition, hyperactive alleles of *ftsA*, *ftsB*, *ftsL*, or *ftsW* can rescue division in the absence of FtsEX (21). All of these methods of rescuing viability probably work by improving assembly and/or function of the septal ring just enough to enable cytokinesis despite the absence of FtsEX (24, 26). Based on this understanding of the phenotypic defect in Δ*ftsEX* strains, we inferred that a selection for multicopy suppressors might lead to the discovery of new division proteins.

## RESULTS

### Isolation of multicopy suppressors of an *ftsEX* null mutation.

We obtained two plasmid libraries from Harris Bernstein (27). The libraries were constructed by partial digestion of E. coli chromosomal DNA with Sau3AI, gel isolation of 2- to 5-kb fragments, and ligation into derivatives of pBR322 (Amp^r^) or pACYC184 (Cam^r^). Because these plasmids are present at about 15 to 20 copies per cell, genes on the inserts should in most cases be overexpressed. This expression would come from promoters present on the inserts, as the vectors did not contain promoters flanking the cloning sites.

Libraries were transformed into strain EC1215 (MG1655 Δ*ftsEX*). A small aliquot of each transformation mixture was plated under permissive conditions (LB Amp or Cam at 30°C) to determine the total number of transformants. The bulk of each transformation mixture was plated under nonpermissive conditions (LB0N Amp or Cam at 37°C) to select for multicopy suppressors. Only 0.18% of the approximately 100,000 transformants plated on LB0N gave rise to a colony, indicating suppressor plasmids are rare. After retransformation to confirm that suppression mapped to the plasmids, DNA inserts were sequenced using primers that bind to vector sequences flanking the cloning site. Paired sequence reads allowed us to infer all of the genes present on the insert by reference to the sequence of the MG1655 genome, except for a few instances where the insert was chimeric.

A total of 87 suppressor plasmids were obtained and characterized. The inserts in these plasmids mapped to 18 distinct chromosomal loci (Table 1). Many of the suppressors were expected, such as *ftsEX* itself and several genes previously reported to function as multicopy suppressors of *ftsEX* null mutations, namely, *ftsN*, *ftsP*, and *dapE* (4, 17, 25). However, some of the suppressor plasmids carried genes of unknown function or genes not previously implicated in cell division. Follow-up studies of one suppressor gene of unknown function, *yedR*, revealed it to be a cell division gene that we have renamed *drpB*.

**TABLE 1:** Multicopy suppressors of the *ftsEX* null mutant

Candidate gene(s) and plasmid(s) (no.)^*a*^	DNA fragment^*b*^		Genes^*c*^
	Left end	Right end	
*ftsEX* (2): pSS12, pSS24	◀3601382	◀2762614	‘*ftsY*→ –*ftsE*→ –*ftsX*→…←*yfjJ*– ←*yfjK*– ←‘*yfjL*
*ftsN* (16 + 32 that were identified by PCR or restriction digestion but not sequenced)			
pSS1	4119775	4121612	‘*cytR*→ –*ftsN*→ –*hslV*’
pSS2	4119953	4122752	‘*priA*→ –*cytR*→ –*ftsN*→ –*hslV*’
pSS10	4119953	4121481	‘*cytR*→ –*ftsN*→ –*hslV*’
pSS16	4119363	4121462	‘*cytR*→ –*ftsN*→ –*hslV*→ –*hslU*’
pSS17, pSS26	4121476	4118459	‘*cytR*→ –*ftsN*→ –*hslV*→ –*hslU*’
pSS27	◀1974935	◀4121828	‘*cytR*→ –*ftsN*→ –*hslV*→…←*cheA*– ←*motB*– ←‘*motA*
pSS48	4121759	4119464	‘*cytR*→ –*ftsN*→ –*hslV*→ –*hslU*’
pSS50	4119464	4122614	‘*cytR*→ –*ftsN*→ –*hslV*→ –*hslU*’
pEP4	4119953	4122614	‘*cytR*→ –*ftsN*→ –*hslV*’
pEP5	4119953	4121543	‘*cytR*→ –*ftsN*→ –*hslV*’
pEP15	4119603	4122857	‘*priA*→ –*cytR*→ –*ftsN*→ –*hslV*→ –*hslU*’
pEP17	4120753▶	4119775▶	‘*cytR*→ –*ftsN*→ –*hslV*’
pEP28	4120052	4121759	‘*cytR*→ –*ftsN*→ –*hslV*’
pEP67	4119953	4123934	‘*priA*→ –*cytR*→ –*ftsN*→ –*hslV*’
pEP69	4119775	4121476	‘*cytR*→ –*ftsN*→ –*hslV*
*ftsP* (*sufI*) (5)			
pEP34	3158158	3161204	‘*plsC*→ –*ftsP*→ –*ygiQ*’
pEP35	3158158	3160870	‘*plsC*→ –*ftsP*→ –*ygiQ*’
pEP51	3158041	3160870	‘*plsC*→ –*ftsP*→ –*ygiQ*’
pEP98	3158695	3160870	‘*plsC*→ –*ftsP*→ –*ygiQ*’
pEP104	3158932	3162021	‘*parC*→ –*plsC*→ –*ftsP*→ –*ygiQ*’
*dapE* (5)			
pSS20	2586927▶	◀1337987	‘*acrD*→ ←*ypfM*– –*yffB*→ –*dapE*→…←*acnA*– ←*ribA*– ←‘*pgpB*
pSS30, pSS37, pSS53	2586927	2591217	‘*acrD*→ ←*ypfM*– –*yffB*→ –*dapE*→ –*ypfN*→ –*ypfH*’
pSS33	2587900	2590862	‘*acrD*→ ←*ypfM*– –*yffB*→ –*dapE*→ –*ypfN*’
*asn* tRNA genes (5)			
pEP23	2042447	2044201	‘*mtA*→ –*asnT*→ –*yeeJ*’
pEP38	2042209	2044201	‘*mtA*→ –*asnT*→ –*yeeJ*’
pEP91, pEP105	2056571	2059465	‘*nac*→ –*cbl*→ ←*asnU*– –*yeeO*’
pEP127	2059462	2061644	‘*cobT*→ –*ldtA*→ ←*asnV* –*nac*’
*rseX* and *drpB* (4)			
pEP1, pEP36	2030722	2032275	*yedS*’– ←*rseX*– –*drpB*→ –*yedJ*’
pEP41	2030635	2032749	*yedS*’– ←*rseX*– –*drpB*→ –*yedJ*’
pEP77	2059462	2061644	*yedS*’– ←*rseX*– –*drpB*→ –*yedJ*→ –*dcm*’
*rodZ* (4)			
pEP11	2639949	2643749	‘*pbpC*→ –*rlmN*→ –*rodZ*’^*d*^
pEP64	2639408	2641391	‘*rlmN*→ –*rodZ*→ –*ispG*’
pEP96	2639431	2641382	‘*rlmN*→ –*rodZ*→ –*ispG*’
pEP99	2639431	2642005	–*ndk*→ ‘*ndk*→ –*rlmN*→ –*rodZ*→ –*ispG*’
*nlpI* (2)			
pEP18	◀941498	◀307499	–*pnp*→ –*nlpI*→ –*deaD*→…*–*rarA*→ –serS*→ –*dmsA*’
pEP60	3305352	3307574	–*pnp*→ –*nlpI*→ –*deaD*→
*accBC* and *yhdT* (2)			
pEP50	3402936	3406522	–*accB*→ –*accC*→ –*yhdT*→ –*panF*’
pEP74	3401725	3406292	‘*acuI*→ –*yrdF*’→^*e*^ –*yrdE*→^*e*^ –*accB*→ –*accC*→ –*yhdT*→ –*panF*’
*pgaCD* (2): pEP79, pEP82	1084845	1088009	‘*pgaB*→ –*pgaC*→ –*pgaD*→ ←‘*phoH*
Various single-copy isolates (8)			
pSS8	2108933	2111754	‘*galF*→ –*rfpB*→ –*rfbD*→ –*rfbA*’
pSS15	1140837	1144074	*rne*’– –*yceQ*→
pSS18	◀4298108	3222397▶	‘*mdtP*→ –*fhdF*→ –*yjcO*→…←*ygjI*– ←*ebgC*– ←‘*ebgA*–
pSS19	2143935	2145995	‘*yegE*→ ←*alkA*– –*yedD*’
pEP9	213131	215019	‘*tilS*→ ←*rof*– ←*yaeP*– –*yaeQ*→ –*arfB*’
pEP26	4008110	4011280	‘*pldB*→ –*yigL*→ –*yigM*→ ←*metR*– ←*metE*’
pEP84	183460	197170	*dxr*’– –*ispU*→ –*cdsA*→ –*rseP*’
pEP86	1159854	11622894	*fhuE*’– –*hinT*→ –*ycfL*→ –*lpoB*→ –*thiK*’

a Grouped by shared candidate genes. The total number of plasmids in each group is in parentheses. Plasmids with identical inserts (i.e., derived from same left and right Sau3AI sites) are separated by commas.

b Numbers refer to base positions of the MG1655 chromosome where digestion with Sau3AI occurred (GenBank accession number U00096). In the case of chimeric inserts, base position is indicated along with an arrowhead to indicate the direction of the fragment (right arrowhead, clockwise; left arrowhead, counterclockwise). See Materials and Methods for further details.

c Genes or gene fragments contained on the inserts, centered on genes common to all inserts. Arrows, dashes, and apostrophes with gene designations have the following meanings: −gene→, a complete gene; ’gene→, the start of the gene is missing (could be a little or a lot missing); –gene', the downstream end of the gene is missing (the apostrophe indicates which end is truncated). Arrows indicate the direction of transcription. Ellipsis dots indicate that sequencing did not continue to the end of the DNA fragment in question, so it is not known what else is on the DNA fragment in that direction.

d Approximately 90% of the *rodZ* gene is present on the pEP11 plasmid.

e *yrdF* and *yrdE* are pseudogenes.

### *drpB* (*yedR*) is a multicopy suppressor of Δ*ftsEX*.

**FIG 1:**
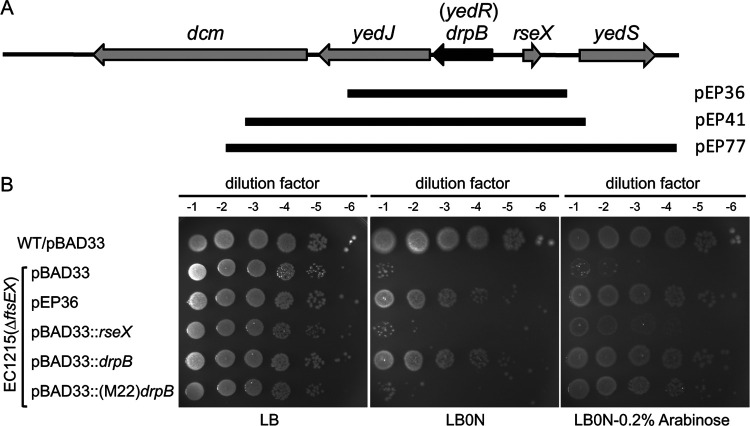
Identification of *drpB* as a multicopy suppressor of Δ*ftsEX*. (A) The E. coli *drpB* locus. Black bars depict the DNA inserts in suppressor plasmids. (B) Plating efficiency of the Δ*ftsEX* mutant EC1215 carrying the suppressor plasmid pEP36 or the indicated derivatives of pBAD33. Overnight cultures were normalized to OD_600_ = 1.0, diluted serially, and spotted (4 μl) onto the indicated plates. Plates were photographed after incubation for 18 h at 37°C. The pBAD33 derivatives are as follows: pBAD33::*rseX* is pDSW1626 and includes 110 bp of chromosomal DNA upstream of the transcriptional start site for *rseX*; pBAD33::*drpB* is pDSW1627 and includes 75 bp of chromosomal DNA upstream of the incorrectly annotated GTG start codon; pBAD33::(M22)*drpB* is pDSW1977, which fuses a synthetic leader sequence and Shine-Dalgarno sequence to the correct translational start site, annotated as ATG-22.

Four of the suppressor plasmids contained the *drpB rseX* locus together with various amounts of flanking DNA (Table 1; Fig. 1A). The plasmid with the smallest insert, pEP36, was characterized further. Transformation of pEP36 into the Δ*ftsEX* null mutant EC1215 improved viability on LB0N plates by about 5 logs compared to a pBAD33 control (Fig. 1B). Plasmid pEP36 also improved cell division in LB0N broth, as evidenced by an average cell length of ∼17 μm for EC1215/pEP36 versus ∼29 μm for EC1215/pBAD33 (see Table S1 in the supplemental material). However, pEP36 only slightly improved growth in LB0N broth (Fig. S1A); in this context it is important to note that EC1215 grows reasonably well in LB0N broth despite having a profound viability defect on LB0N plates. The *rseX* gene encodes a small RNA of 91 nucleotides that downregulates production of two outer membrane proteins, OmpA and OmpC, and affects the sigmaE-dependent response to outer membrane stress (28). DrpB was annotated as YedR, a small, nonessential inner membrane protein with a GTG start codon and no known function (29–32). To determine whether *rseX* or *drpB* was the relevant suppressor, we cloned each gene separately into pBAD33, a vector with the arabinose-inducible P_BAD_ promoter (33). We found that pBAD33::*drpB* but not pBAD33::*rseX* rescued EC1215 on LB0N plates (Fig. 1B). pBAD33::*drpB* also improved both growth and division when EC1215 was grown in LB0N broth (Fig. S1B; Table S1). Multicopy *drpB* did not rescue *ftsZ*(Ts), *ftsA*(Ts), *ftsQ*(Ts), or *ftsI*(Ts) mutants (Fig. S1C). *In toto*, these findings indicated that *drpB* is a multicopy suppressor of Δ*ftsEX* but is not broadly capable of suppressing division defects *per se*.

### DrpB translation initiates primarily at an ATG annotated as codon 22.

**FIG 2:**
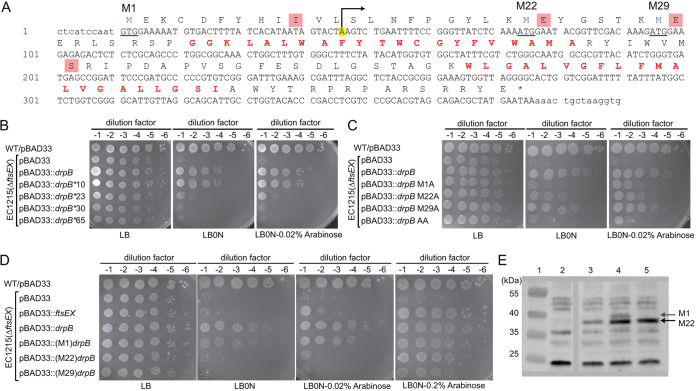
Mapping the *drpB* translational start site and promoter. (A) DNA and predicted amino acid sequence of *drpB* as annotated in EcoCyc. YedR (DrpB) transmembrane domains are in red lettering. The annotated translational start site is labeled M1. Other potential translational start sites are designated M22 and M29. Amino acids encased in red boxes were changed to stop codons. A previously mapped transcriptional start site is highlighted yellow and marked with an arrow. (B) Viability assay to test rescue by pBAD33::*drpB* derivatives with stop codons at the indicated sites. The pBAD33 derivatives were pDSW1627, pDSW1643, pDSW1914, pDSW1916, and pDSW1918. (C) Viability assay to test rescue by pBAD33::*drpB* derivatives with the indicated Met codons mutated to alanine. The pBAD33 derivatives were pDSW1627, pDSW1930, pDSW1931, pDSW1940, and pDSW1955. (D) Viability assay to test rescue by DrpB proteins engineered to direct translational initiation to M1, M22, or M29. The pBAD33 derivatives were pDSW610, pDSW1627, pDSW1975, pDSW1977, and pDSW1979. (E) Western blot of DrpB-GFP probed with polyclonal anti-GFP serum. Lane 1, size markers, with masses indicated to the left of the blot; lane 2, EC251 (WT with no GFP fusion); lane 3, EC4680 (chromosomal *drpB-gfp* at native locus); lane 4, EC251/pDSW1642 [P_206_::(M1)*drpB-gfp*]; lane 5, EC251/pDSW1934 [P_206_::(M22)*drpB-gfp*]. Arrows point to the M1 and M22 forms of DrpB-GFP. Note that despite changing GTG-1 to ATG and providing a synthetic Shine-Dalgarno sequence, translation initiates preferentially at the internal M22 start site that lacks an obvious Shine-Dalgarno sequence.

Curiously, pBAD33::*drpB* rescued Δ*ftsEX* even in the absence of the inducer arabinose (Fig. 1B). Furthermore, preventing *drpB* expression by mutating the codon for Ile 10 to a stop codon did not abrogate rescue (Fig. 2B). These observations prompted us to investigate whether *drpB* had been annotated properly. According to EcoCyc release 20.1 (and many other databases), *drpB* is predicted to encode a 121-amino-acid protein with a GTG start codon and two transmembrane helices (Fig. 2A) (29). The basis for inferring the GTG start codon was unclear, as it lacks an obvious Shine-Dalgarno sequence. Inspection of the DNA sequence revealed alternative potential start codons at ATG-22 and ATG-29, although neither of these had a promising Shine-Dalgarno sequence either.

To identify the correct start codon, we constructed pBAD33::*drpB* variants with stop codons at positions 23 and 30. Neither of these plasmids rescued the Δ*ftsEX* mutant on LB0N (Fig. 2B). This result suggested the start codon is ATG-22, but in that case changing ATG-22 to Ala should have prevented rescue, which it did not (see pBAD33::*drpB* M22A in Fig. 2C). Further site-directed mutagenesis revealed that both GTG-1 and ATG-22 had to be changed to alanine codons simultaneously to abrogate rescue (see pBAD33::*drpB* AA in Fig. 2C). Thus, at least when expressed from a plasmid, translation of *drpB* can initiate at either GTG-1 or ATG-22 to produce a 121-amino-acid protein that we will call (M1)DrpB or a 100-amino-acid protein that we will call (M22)DrpB.

To identify the preferred translational start site when *drpB* is expressed from its native chromosomal locus, we turned to Western blotting. Because we had antibody against GFP but not DrpB, we used lambda Red technology to fuse *gfp* via a 5-amino-acid linker to the 3′ end of *drpB in situ* (34). The resulting DrpB-GFP fusion protein should be 41.3 kDa or 38.8 kDa, depending on whether translation initiates at GTG-1 or ATG-22. For a direct comparison, we cloned *drpB* into the GFP fusion vector pDSW210, which expresses target genes under the control of a weak IPTG-inducible promoter designated P_206_ (35). Two derivatives of pDSW210 were made. In the first, GTG-1 was changed to ATG and furnished with a strong, synthetic Shine-Dalgarno sequence to direct production of (M1)DrpB-GFP. In the other plasmid, the synthetic Shine-Dalgarno sequence was linked to ATG-22 to produce (M22)DrpB-GFP.

Western blotting with anti-GFP serum revealed only (M22)DrpB-GFP when *drpB-gfp* was expressed from the native chromosomal locus (Fig. 2E). The preference for ATG-22 over GTG-1 as a start site is underscored by the finding that even the plasmid engineered to produce (M1)DrpB-GFP produced instead mostly (M22)DrpB-GFP by a ratio of 5:1. This result is surprising, given that we cannot identify a very credible Shine-Dalgarno sequence upstream of ATG-22. We conclude that DrpB is a 100-amino-acid protein that initiates at the ATG annotated as codon 22. For clarity and consistency, we will continue to use the names (M1)DrpB and (M22)DrpB throughout the article, although M22 is in reality the first amino acid.

### Promoter for *drpB* is between the codons annotated as GTG-1 and ATG-22.

The pBAD::*drpB* plasmid (pDSW1627) used for the rescue experiment shown in Fig. 1B included 75 base pairs (bp) of DNA upstream of the GTG codon thought at the time to be the translational start. Curiously, this plasmid rescued the Δ*ftsEX* mutant on LB0N even in the absence of arabinose (Fig. 1B), suggesting the cloned insert fortuitously included the *drpB* promoter. Consistent with this inference, according to RegulonDB a transcription start site has been mapped by transcriptome sequencing (RNA-seq) between GTG-1 and ATG-22 (highlighted in yellow in Fig. 2A) (36). We therefore built a new set of pBAD33::*drpB* plasmids in which a synthetic Shine-Dalgarno sequence was linked to GTG-1, ATG-22, or ATG-29 to produce proteins designated M1, M22, and M29, respectively. All three plasmids rescued the Δ*ftsEX* mutant (Fig. 2D). Rescue was independent of arabinose for the M1 construct, but required arabinose for the M22 and M29 constructs. We conclude that the promoter for *drpB* is located between GTG-1 and ATG-22 (as originally indicated by RNA-seq) and that the first seven amino acids of DrpB are not required for rescue of Δ*ftsEX* because the truncated M29 form of DrpB was likewise capable of rescue.

### DrpB is a septal ring protein.

**FIG 3:**
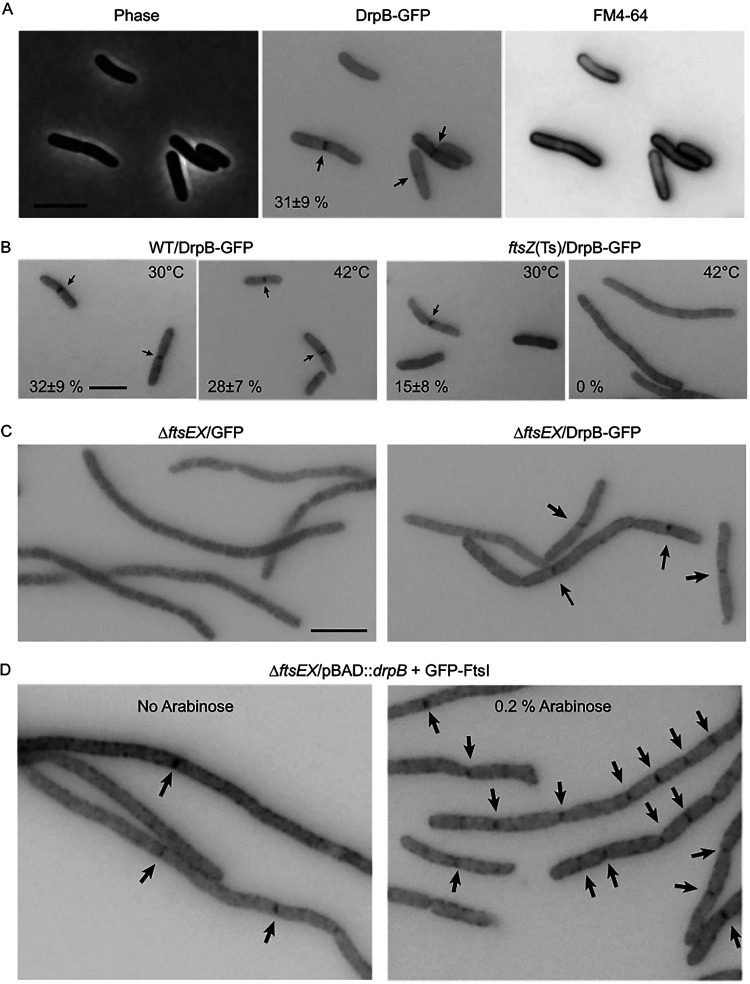
DrpB is a septal ring protein and improves recruitment of FtsI in the absence of FtsEX. (A) DrpB-GFP localizes to the division site. The WT strain EC251/pDSW1934 [P_206_::(M22)*drpB-gfp*] was grown at 30°C in LB0N. Membranes were visualized by staining with FM4-64. Fluorescence images were inverted to better visualize GFP (arrows). (B) Septal localization of DrpB requires FtsZ. The WT strain EC251 and the *ftsZ84*(Ts) mutant EC309 were transformed with pDSW1934 [P_206_::(M22)*drpB-gfp*]. Cultures were grown in LB at 30°C before shifting to LB0N at 42°C for 30 min. (C) Septal localization of DrpB does not require FtsEX. Transformants of EC1215 (Δ*ftsEX*) carrying pDSW210 [P_206_::*gfp*] or pDSW1934 [P_206_::(M22)*drpB-gfp*] were grown in LB0N. (D) Overproduction of DrpB partially restores septal localization of FtsI in the absence of FtsEX. EC4762 [Δ*ftsEX*/pDSW1977 {P_BAD_::(M22)*drpB*} and pDSW235 (P_206_::*gfp-ftsI*)] was grown at 30°C in LB0N containing 100 μM IPTG (to induce *gfp-ftsI*) and without or with 0.2% arabinose (to induce *drpB*). When cultures reached OD_600_ = 0.3, cells were fixed and imaged. Arrows point to septal localization of GFP-FtsI. Percentages in panels A and B indicate the fraction of cells exhibiting septal localization (mean ± standard deviation [SD] from at least two experiments). Size bars = 5 μm. Images in panels C and D are representative of two experiments.

To test for septal localization, we used the above-mentioned DrpB-GFP fusion plasmids. The fusions were stable as assessed by Western blotting (Fig. 2E) and functional as assessed by complementation (see “Δ*drpB* Δ*dedD* double mutant is filamentous” below). When a wild-type (WT) strain harboring pDSW1934 [P_206_::(M22)*drpB-gfp*] was grown in LB0N, about 30% of cells exhibited a weak fluorescent band at the midcell, indicating that DrpB-GFP localizes to the septal ring (Fig. 3A; Table 2). Staining membranes with FM4-64 verified that septal localization of DrpB-GFP preceded constriction and was not an artifact of there being two membranes at the site of invagination (Fig. 3A). Septal localization was also observed when a *drpB-gfp* fusion was produced from the native chromosomal locus (Fig. S2; Table 2), although in this case the fluorescent signals were even weaker. As expected, septal localization of DrpB-GFP required FtsZ but not FtsEX (Fig. 3B and C). The difficulty of visualizing DrpB-GFP fusions dissuaded us from characterizing localization dependences in detail.

**TABLE 2:** Septal localization frequencies of DrpB fusions to GFP

Description of fusion	Plasmid name	No. of linker aa^*a*^	Localization frequency (%)^*b*^	
			WT background	Δ*drpB* mutant
P_206_::(M1)*drpB-gfp*	pDSW1642	5	31 ± 2 (*n* = 2)	ND
P_206_::(M22)*drpB-gfp*	pDSW1934	5	31 ± 9 (*n* = 2)	27 ± 8 (*n* = 7)
P_204_::(M1)*drpB-gfp*	pDSW1901	64	11 ± 8 (*n* = 2)	ND
P_206_::*gfp*-(M22)*drpB*	pDSW1991	7	ND	15 ± 7 (*n* = 3)
*drpB-gfp* at native chromosomal locus	Not applicable	5	24 ± 4 (*n* = 3); *drpB-gfp* is the only *drpB* allele present	

a aa, amino acids.

b Percentage of cells scored positive for septal localization (mean ± SD from the indicated number of trials). Cultures were grown in LB0N at 30°C. ND, not determined.

We made several attempts to overcome the weak fluorescent signals provided by (M22)DrpB-GFP. Thinking that perhaps modification of the C terminus of DrpB interferes with septal localization, we constructed a GFP-(M22)DrpB fusion, but it too localized weakly (Fig. S3; Table 2). Other efforts to improve localization included changing the amount of IPTG used for induction, lengthening the linker, expressing fusions in a Δ*drpB* background, fixing cells with paraformaldehyde, and growing cells at temperatures ranging from room temperature to 42°C. None of these efforts were fruitful (Table 2 and data not shown).

### Overproduction of DrpB improves divisome assembly in a Δ*ftsEX* mutant.

Multicopy suppression by DrpB presumably works by improving recruitment of downstream division proteins despite the absence of FtsEX. To test this notion, we introduced two plasmids into a Δ*ftsEX* mutant. One plasmid was a pBAD33 derivative that allowed for overexpression of (untagged) *drpB* under the control of an arabinose-inducible promoter, while the other was a pDSW210 derivative that expressed *gfp-ftsI* from an IPTG-inducible promoter. As expected, addition of arabinose to the culture medium to drive overexpression of *drpB* improved localization of GFP-FtsI (Fig. 3D). We conclude that overproduction of DrpB rescues a Δ*ftsEX* mutant in LB0N by improving recruitment of “downstream” division proteins required for synthesis of septal PG. This conclusion is consistent with previous reports of Δ*ftsEX* suppression (21, 24, 37). Nevertheless, it is worth noting that the Δ*ftsEX* mutant was still filamentous when DrpB was overproduced in our experiments. This is because (i) *drpB* is not a strong suppressor, (ii) overproduction of DrpB might not recruit the cell wall amidases (23), and (iii) GFP fusions to Fts proteins actually inhibit division when expressed in the Δ*ftsEX* background, even fusions like GFP-FtsI that appear to be fully functional in the context of an otherwise healthy division apparatus (24, 35).

### Septal localization of DrpB is strongly dependent on growing E. coli in low-osmotic-strength medium.

**FIG 4:**
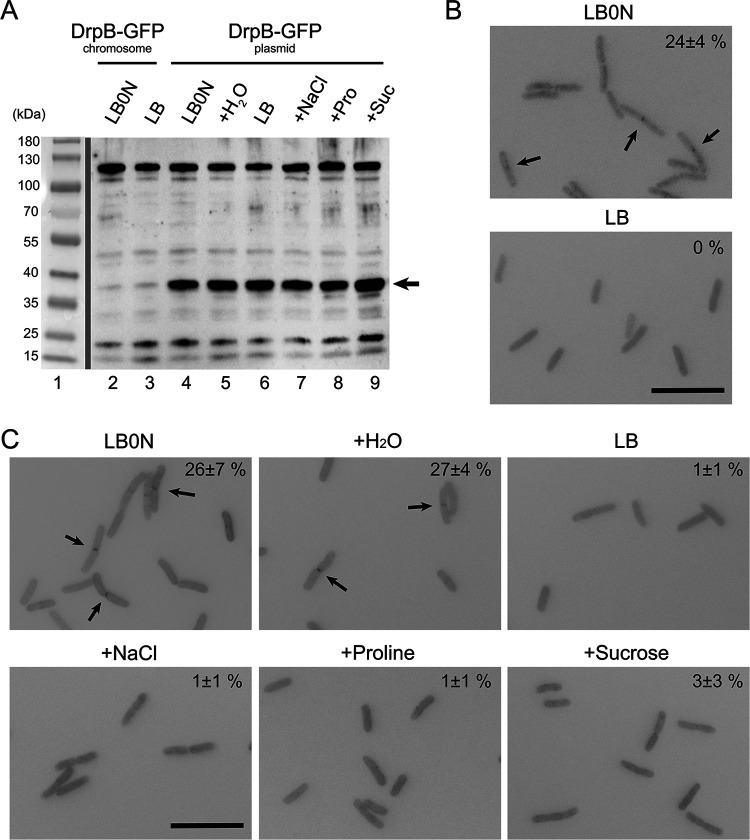
Elevated osmotic strength impairs septal localization of DrpB. (A) Western blot with polyclonal anti-GFP serum. The arrow indicates DrpB-GFP. The identity of the strong band at 120 kDa is not known. (B) Septal localization of DrpB-GFP expressed from the native chromosomal locus in strain EC4680. (C) Septal localization of plasmid-expressed DrpB-GFP in a Δ*drpB* background (EC4796). In the micrographs arrows point to septal localization and numbers refer to the fraction of cells scored positive for septal localization (mean ± SD from two experiments). Size bar = 10 μm. For this experiment, overnight cultures grown in LB were diluted into LB or LB0N; where indicated, LB0N was supplemented with NaCl, proline, or sucrose to a 200 mM final concentration.

During the course of our localization experiments, we stumbled across the remarkable observation that septal localization of DrpB-GFP is almost completely dependent on growing cells in LB0N. For example, in a set of experiments shown in Fig. 4, (M22)DrpB-GFP localized in ∼25% of the cells grown in LB0N compared to only 1% of cells grown in LB. Despite this difference, the fusion proteins were expressed at similar levels in both media (Fig. 4; Fig. S3). A GFP-(M22)DrpB fusion behaved similarly (Fig. S3), arguing the phenomenon is an intrinsic property of DrpB rather than an artifact introduced by the GFP tag. The only difference between LB and LB0N is that the former contains NaCl at 10 g/liter, equivalent to ∼170 mM. To explore the role of NaCl in inhibiting localization of DrpB, we tested LB0N amended with 200 mM NaCl, proline, or sucrose. All three additives essentially abrogated septal localization of (M22)DrpB-GFP (Fig. 4). Thus, poor localization in LB is a consequence of the high osmotic strength rather than a direct inhibitory effect of NaCl *per se*.

### Δ*drpB* Δ*dedD* double mutant is filamentous.

**FIG 5:**
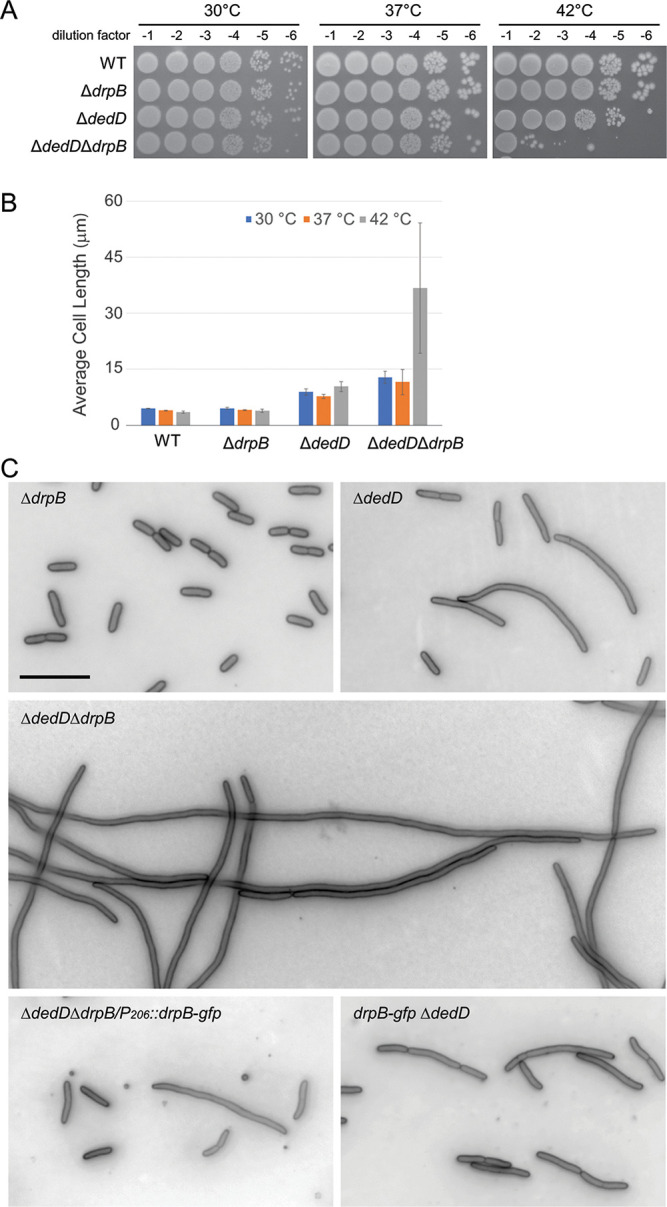
A Δ*drpB* Δ*dedD* mutant has a profound division defect when grown at elevated temperature in LB. (A) Plating efficiency on LB. (B) Cell length when grown in LB broth (mean ± SD from two experiments). (C) Representative micrographs of cells grown in LB at 42°C and stained with FM4-46. Size bar = 10 μm. Strains shown are EC251, EC4622, EC4774, EC4775, and EC4775/pDSW1934 [P_206_::(M22)*drpB-gfp*].

We did not observe any noteworthy viability or division defects when a Δ*drpB* mutant was grown in LB, LB0N, or M9 minimal medium (shown for LB in Fig. 5). To look for synthetic phenotypes, we used P1 transduction to combine Δ*drpB* with mutations in several other division genes. The resulting double mutants were tested for viability on LB and LB0N at various temperatures (Fig. S4). Only one striking synthetic phenotype was uncovered: a Δ*drpB* Δ*dedD* double mutant exhibited a 3-log drop in plating efficiency on LB at 42°C (Fig. 5A) and became filamentous when grown in LB broth at 42°C (Fig. 5B and C). Staining with FM4-64 revealed few septa, indicating the primary defect is at the level of constriction rather than separation of daughter cells (Fig. 5C).

The profound filamentation defect of the Δ*drpB* Δ*dedD* double mutant allowed us to demonstrate that our (M22)DrpB-GFP fusion is functional. First, producing (M22)DrpB-GFP from a plasmid corrected the synthetic defect of the double mutant back to the more modest defect of a single Δ*dedD* mutant (Fig. 5C). Second, we used P1 transduction to delete *dedD* in a *drpB-gfp* background. The resulting *drpB-gfp* Δ*dedD* strain divided like a simple Δ*dedD* mutant rather than like Δ*drpB* Δ*dedD* double mutant (Fig. 5C).

### Bioinformatic analysis of DrpB.

**FIG 6:**
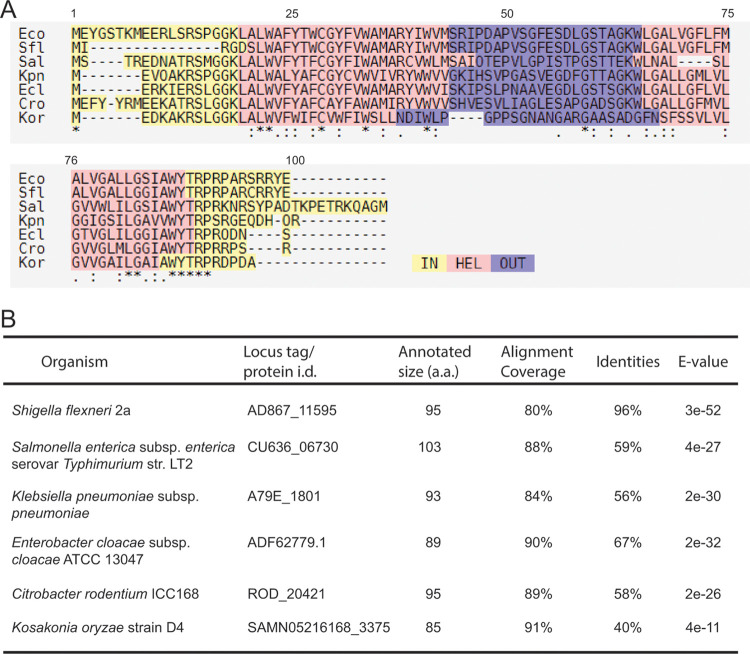
Conservation of DrpB homologs. (A) Multiple sequence alignment produced by PSI/TM-Coffee. Predicted cytoplasmic domains are shaded yellow, transmembrane helices are pink, and the periplasmic loop is purple. (B) Homologs of E. coli DrpB used to produce the alignment.

Searches conducted with Tblastn (38) revealed that DrpB is not widely conserved. We found homologs in a subset of *Gammaproteobacteria*, including multiple E. coli strains, Shigella flexneri, Salmonella enterica serovar Typhimurium, and Klebsiella pneumoniae (Fig. 6). However, we failed to identify homologs in other *Gammaproteobacteria* such as Citrobacter freundii, Pseudomonas aeruginosa, Serratia marcescens, Vibrio cholerae, and Yersinia pestis. Nor could we identify homologs by searching the genomes of more distantly related bacteria often used to study cell division, such as Caulobacter crescentus, Myxococcus xanthus, Bacillus subtilis, or Staphylococcus aureus.

A search of the Pfam database release 32.0 (September 2018) failed to turn up any conserved domains, even domains of unknown function (39). To look for conserved features of DrpB, we used T-Coffee (40) to generate a multiple sequence alignment (Fig. 6). DrpB proteins are short, ranging from 85 to 103 amino acids. Overall sequence conservation is weak but the protein’s architecture is well conserved: both the N and C termini are in the cytoplasm and there are two transmembrane helices (TMH), which are separated by a periplasmic loop of ∼20 amino acids. This predicted topology agrees with that determined in a large-scale topological analysis of E. coli membrane proteins (32). Sequence conservation is strongest in the TMHs, especially the distal end of TMH2.

### DrpB interacts with multiple proteins in a bacterial two-hybrid system.

DrpB presumably localizes to the divisome by binding directly to one or more divisome proteins. To screen for interaction partners, we used the bacterial adenylate cyclase two-hybrid (BACTH) system, which has been used in many studies of protein-protein interactions involved in divisome assembly (10, 11, 41–46). Briefly, we fused the T18 fragment of Bordetella pertussis adenylate cyclase to the C terminus of DrpB in an Amp^r^ plasmid with a pBR origin of replication and a copy number of ∼20. This plasmid was paired with T25 fusions to 13 divisome proteins produced from a Kan^r^ plasmid with a p15A origin and a copy number of ∼15. As negative controls, we paired the DrpB-T18 plasmid with the T25 empty vector and with T25 fusions to three membrane proteins not involved in cell division: the elongation-specific peptidoglycan synthase PBP2 and the maltose transporter membrane proteins MalF and MalG (47, 48).

**FIG 7:**
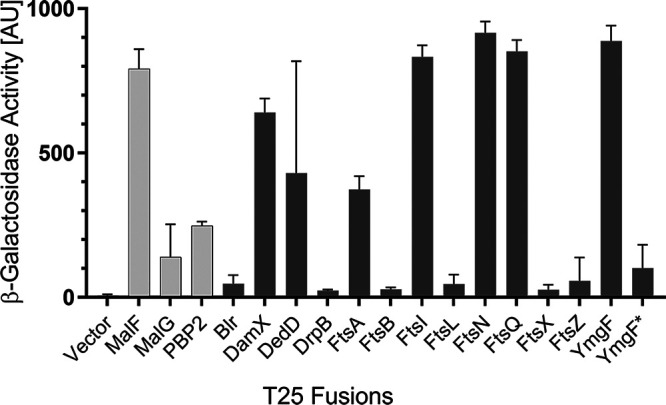
BACTH analysis of DrpB interaction with division proteins. The T18 domain of adenylate cyclase was fused to DrpB. The T25 domain of adenylate cyclase was fused to the proteins indicated; all fusions are in pKT25 except for YmgF*, which is in pKNT25. Fusions were coexpressed in E. coli strain DHM1 and β-galactosidase activities were measured as a proxy for functional reconstitution of adenylate cyclase. Light gray bars, negative controls; dark gray bars, divisome proteins. Values depict the mean ± SD from four independent experiments. The unexpected interactions with MalF and PBP2 were confirmed using new transformants after verification of the plasmids by restriction digestion.

Quantitative β-galactosidase assays indicated DrpB interacts strongly with DamX, FtsI, FtsN, FtsQ, YmgF (one configuration only), and the negative control MalF (Fig. 7). Weaker interactions were observed with DedD, FtsA, and the two remaining negative controls MalG and PBP2. Little or no interaction was observed with Blr, DrpB itself, FtsB, FtsL, FtsX, FtsZ, or with the soluble T25 fragment produced from the empty vector.

Further studies will be needed to ascertain the biological relevance of any of the observed interactions, many of which are likely to be false positives. We are not the first to encounter issues with an implausibly high number of interaction partners using the BACTH system. For example, according to published studies, the 41-amino-acid membrane protein Blr interacts with seven of 12 divisome proteins tested, the 72-amino-acid membrane protein YmgF interacts with all nine divisome proteins tested, and B. subtilis FtsEX interacts with 19 out of 20 B. subtilis membrane proteins tested (10, 11, 49). We suspect the T18 and T25 fragments of *Bordetella* adenylate cyclase retain sufficient affinity for one another that tethering them to the membrane sometimes raises their local concentration sufficiently to drive reconstitution directly. If so, empty vectors that produce soluble T18 or T25 fragments are not adequate negative controls for assaying membrane protein interactions with the BACTH system.

## DISCUSSION

### Multicopy suppressors of FtsEX fall into three classes.

We obtained 87 suppressor plasmids that rescue growth of an E. coli Δ*ftsEX* mutant on low osmotic strength medium, where the mutant is not viable owing to inefficient recruitment of so-called late (or downstream) divisome proteins required for cytokinesis (21, 24). These suppressor plasmids can be divided into three classes based on the type of genes they carry.

### (i) Class I plasmids.

Class I plasmids overproduce division proteins. This class accounts for 74% of the plasmids recovered in our selection. The genes on these plasmids include *ftsEX*, *ftsN*, *ftsP*, *dapE*, *drpB*, *lpoB*, and *rof* (4, 17, 24, 25, 50–53). The first six of these encode divisome proteins. The lone exception, *rof*, codes for an antiterminator that promotes overproduction of multiple division proteins and is a known multicopy suppressor of a variety of *fts* mutations (54). Overproduction of divisome proteins presumably drives assembly by mass action. This mechanism of suppression has been explored in detail by Lutkenhaus and coworkers, who identified a pivotal role for FtsN-mediated “back recruitment” in facilitating divisome assembly in the absence of FtsEX (26, 37). Further support comes from the present study in which we showed overproduction of DrpB improves septal localization of the “downstream” PG synthase FtsI (Fig. 3).

### (ii) Class II plasmids.

Class II plasmids overproduce proteins that, while not canonical divisome proteins, nevertheless play roles in synthesis or remodeling of cell envelope: proteins encoded by *rodZ*, *nlpI*, *ispU*, *accBC*, *rfbBD*, and *pagCD* (29). The ability of such plasmids to rescue a Δ*ftsEX* mutant can be rationalized by the fact that elongation and division are linked by shared morphogenic proteins, enzymes, lipid carriers, and precursors (55–57). There are a few reports of elongation proteins interacting with division proteins or localizing transiently to the midcell (58–62). In addition, changes in cell shape can affect the operation of pathways for envelope biogenesis (63). That said, the connections between elongation and division are poorly understood, so further studies will be required to explain how class II suppressors can rescue septal ring assembly in the absence of FtsEX.

### (iii) Class III suppressors.

Class III suppressors comprise genes without any obvious connection to cell envelope processes. These suppressors, which tend to be weak, are the most difficult to rationalize, as exemplified by plasmids coding for three different asparagine tRNAs that recognize GUU codons. One can speculate that overproducing these tRNAs increases expression of a pivotal division or elongation gene. However, the most obvious candidate, *ftsN*, does not have a single GUU codon.

Our selection for multicopy suppressors of Δ*ftsEX* returned the various division genes with strikingly different frequencies. We think these differences can be explained by the relative potency of various suppressor genes and the frequency sites for Sau3AI, the restriction enzyme used to prepare chromosomal DNA for the plasmid libraries (27). Over half the suppressor plasmids carried *ftsN*, a strong suppressor that plays a pivotal role in driving divisome assembly in the absence of FtsEX (26). Moreover, *ftsN* has only one Sau3AI site and its promoter is immediately upstream of the gene. In contrast, only two of our 87 suppressor plasmids carried *ftsEX* and these are probably siblings, seeing as both had the same chimeric insert. The scarcity of *ftsEX* plasmids is, at first glance, surprising because the simplest way to rescue a Δ*ftsEX* mutant would be to provide a functional copy of *ftsEX* in *trans*. But *ftsEX* has nine Sau3AI sites and the promoter is over 1.5 kb away, upstream of *ftsY*, which adds three more Sau3AI sites.

### Implications for models of divisome assembly.

The class I plasmids rescue division by rescuing septal ring assembly, as shown here (Fig. 3D) and in previous studies of Δ*ftsEX* suppression (21, 24, 26). The ability to drive septal ring assembly by overproducing any of several division proteins lends further support to models that depict divisome assembly as involving a network of protein interactions over models that emphasize a more linear set of binary interactions, as was suggested by early localization-dependency studies (2, 3, 64–66). Why might natural selection favor a network? Networks are relatively robust to perturbations because when one link fails, other links can be strengthened to compensate. Linear pathways, by comparison, are more susceptible to catastrophic failure because they only function when every step works just right. Another important difference is that networks are well suited for integrating information from many sources into a decision-making process, in this case the decision of whether to divide. Linear pathways, on the other hand, are well designed for regulating processes with simple checkpoints. Similar considerations might also explain why the divisome contains so many nonessential proteins, which by now outnumber the essential ones by more than two to one. For an organism that lives in a perilous and unpredictable world, the flexibility afforded by a highly networked and adaptable division apparatus might be preferable to a precisely engineered machine with no superfluous parts and little tolerance for error.

### DrpB.

Perhaps the most important finding to emerge from our multicopy suppressor selection is the discovery of a new component of the E. coli division apparatus, DrpB. DrpB appeared under our selection because it localizes to division sites and rescues septal ring assembly when a Δ*ftsEX* mutant is grown under nonpermissive conditions (Fig. 3). Homologs of DrpB are found only in E. coli and its closest relatives, and the protein lacks any conserved domains, even domains of unknown function. Although deleting *drpB* has little or no effect on division, a Δ*drpB* Δ*dedD* double mutant has a severe division defect on LB, especially at elevated temperatures. Fusions of DrpB to GFP (both DrpB-GFP and GFP-DrpB) exhibited faint but unambiguous septal localization in up to 30% of the cells when E. coli was grown in LB0N. Curiously, very little septal localization was observed in LB, or in LB0N amended with 200 mM NaCl, sucrose, or proline. Thus, DrpB localization is impaired by high osmolarity rather than high salt *per se*.

Although we have observed over the years that many GFP-Fts fusions localize somewhat better when E. coli is grown in LB0N than in LB, DrpB might provide the first example of a division protein whose localization is almost completely dependent upon low osmotic strength. Several *Caulobacter* PG synthesis proteins exhibit osmolarity-dependent changes in localization (67). However, the *Caulobacter* proteins accumulate at the division site upon shift to high osmolarity, not low, and the response in *Caulobacter* is transient, as the proteins in question redistribute from the division site to the cell cylinder as the organism adapts over a period of one to two generations (67).

Further work will be needed to determine the precise role of DrpB in E. coli cell division. The simple-minded notion that DrpB serves to fortify the divisome under conditions of low osmolarity is probably wrong because *drpB* expression is not induced by low salt (Fig. 4) and because the severe division defect in a Δ*drpB* Δ*dedD* double mutant was observed in LB but not in LB0N (Fig. 5). The recent discovery that DedD activates synthesis of septal PG (46) suggests that *drpB* is somehow involved in this process as well, perhaps by improving FtsN activity (21, 26).

## MATERIALS AND METHODS

### Media.

Lysogeny broth (LB) consisted of 10 g tryptone, 5 g yeast extract, and 10 g NaCl per liter, with 15 g agar/liter for plates. LB0N was the same except that NaCl was omitted. Antibiotics were used at the following concentrations: ampicillin, 200 μg/ml; chloramphenicol, 30 μg/ml for plasmids and 15 μg/ml for chromosomal markers; kanamycin, 40 μg/ml. l-Arabinose was added at 0.2% where indicated to induce expression of genes under P_BAD_ control. Isopropyl-β-d-1-thiogalactopyranoside (IPTG) was used at 25 μM to induce *gfp* fusions to *drpB* and at 100 μM to induce *gfp-ftsI*.

### Bacterial strains, oligonucleotide primers, and plasmids.

Standard procedures were used for analysis of DNA, PCR, electroporation, transformation, P1 transduction, and lambda Red recombineering (34, 68, 69). Bacterial strains are listed in Table S2 in the supplemental material, which also describes how these strains were made. Plasmids are described in Table S3, followed by descriptions of how these plasmids were made. Oligonucleotides were from Integrated DNA Technologies (Coralville, IA), and are listed in Table S4. Enzymes were from New England BioLabs (Ipswich, MA). Regions of plasmids constructed by PCR were verified by DNA sequencing at the DNA Core Facility of the Carver College of Medicine using dye-termination cycle-sequencing technology.

### Selection of multicopy suppressors.

Plasmid libraries were transformed into EC1215 chemically competent cells. An aliquot of the transformation mixtures was plated on LB with Amp (for the pBR library) or LB with Cam (for the pACYC library) and incubated 16 h at 37°C to determine the total number of transformants. The bulk of each transformation mixture was plated on LB0N with Amp or Cam and incubated 16 h at 37°C to select for suppressors. The resulting colonies were recovered by streaking onto LB with antibiotic at 30°C and then tested by streaking onto LB0N with antibiotic at 37°C. Candidates that showed the suppressor phenotype upon retesting were characterized further. Plasmid DNA was isolated from each candidate and retransformed into EC1215, selecting on LB with antibiotic at 30°C. Transformants were purified by streaking on LB with antibiotic at 30°C and then tested for the suppressor phenotype by streaking onto LB0N with antibiotic at 37°C. The inserts on validated suppressor plasmids were sequenced from each end using primers P959 and P960 (pBR-based suppressors) or P971 and P972 (pACYC-based suppressors). Sequence reads were mapped to the E. coli K-12 MG1655 genome (GenBank accession number U00096) using BLAST in 2006 using the EcoGene website (70). EcoGene no longer exists. Sequences for DNA inserts in suppressor plasmids can currently be retrieved using the fragment endpoints listed in Table 1 to search the correct MG1655 genome sequence in PATRIC (71).

### Viability testing.

Unless indicated otherwise in the figure legend, overnight cultures were grown at 30°C in LB containing antibiotic as appropriate. In the morning, cells from 1 ml of culture were harvested by centrifugation and the cell pellet was suspended in LB or LB0N to achieve an optical density at 600 nm (OD_600_) of 1.0. Serial dilutions (10-fold) were made in LB or LB0N before spotting 3 μl onto LB or LB0N plates containing antibiotics and, where indicated, arabinose. Plates were photographed after incubating for 18 h at the indicated temperature.

### Effect of pEP36 and pBAD33::*drpB* on growth and division in broth cultures.

Overnight cultures of EC1215/pBAD33 and EC1215/pEP36 grown in LB Cam at 30°C were diluted 1:200 into 22 ml of LB0N Cam in 250-ml baffle flasks and cultures were grown at 30°C and 210 rpm. Growth was monitored by measuring OD_600_. When the culture density reached OD_600_ of 0.4 to 0.5, cells were photographed under phase-contrast microscopy. Average cell length was determined by measuring at least 100 cells per experiment. The effect of pBAD33::*drpB* on growth and division was determined similarly except that overnight cultures were diluted 1:200 into LB0N Cam containing 0.2% l-arabinose to induce the P_BAD_ promoter.

### Western blotting.

Overnight cultures were grown at 30°C in LB containing antibiotic as appropriate. In the morning, cells were diluted 1:200 into the indicated medium and grown at the indicated temperature to OD_600_ = ∼0.5. Cells from 1 ml of culture were harvested by centrifugation and the cell pellet was taken up in 100 μl of 1× Laemmli sample buffer (63 mM Tris-HCl [pH 6.8], 10% glycerol, 2% sodium dodecyl sulfate, 0.0005% bromophenol blue, 0.1% 2-mercaptoethanol). Samples were heated for 10 min at 95°C before loading 10 μl onto a precast mini-PROTEAN TGX gel (10% polyacrylamide) (Bio-Rad, Hercules, CA). Electrophoresis, transfer to nitrocellulose, and blot development followed standard procedures. Blocking was with 5% nonfat dry milk diluted in PBST (phosphate-buffered saline containing 0.1% TWEEN 20). Primary antibody was polyclonal rabbit anti-GFP serum diluted 1:10,000 in PBST. Secondary antibody was horseradish peroxidase-conjugated goat anti-rabbit antibody (1:8,000) (Pierce, Rockford, IL), which in turn was detected with SuperSignal Pico West PLUS chemiluminescent substrate (Thermo Scientific, Rockford, IL). Blots were visualized with a ChemDoc Touch imaging system (Bio-Rad, Hercules, CA).

### Anti-GFP antiserum.

Polyclonal antibodies against GFP were raised in New Zealand White rabbits (ProsSci, San Diego, CA). The protein used as antigen was obtained as follows. His_6_-GFP was overproduced in BL21(DE3)/pDSW1883 and purified by Talon cobalt affinity chromatography (TaKaRa, Mountain View, CA) according to instructions from the manufacturer. Purified His_6_-GFP was dialyzed into 50 mM NaPO_4_ (pH 7.5), 150 mM NaCl. The preparation was >95% pure with a concentration of 1.2 mg/ml. An aliquot to be used for raising antibodies was dialyzed against water before shipping to ProSci. Prior to use, anti-GFP serum was incubated with a lysate of E. coli EC251 to reduce cross-reaction with cellular proteins (72).

### Growth of cultures for microscopy.

Starter cultures were grown overnight at 30°C in LB containing Kan or Amp as appropriate.

For Fig. 3A, overnight culture was diluted 1:200 into LB0N Amp IPTG (25 μM). Cultures were grown at 30°C to OD_600_ = 0.3, at which time samples were taken for microscopy, stained with FM4-64, spotted on an agarose pad, and photographed as indicated. Subsequent experiments followed a similar sequence of steps except as follows. For Fig. 3B, cultures were grown at 30°C in LB Amp to OD_600_ = 0.5, then diluted 1:20 into LB0N Amp IPTG at 30°C or 42°C for 30 min prior to capturing micrographs of live cells immobilized on agarose pads. For Fig. 3C, overnight cultures were diluted 1:200 into LB0N Amp IPTG and grown at 30°C for about 4 h to OD_600_ = 0.3 prior to microscopy. This experiment was done twice with live cells and twice with cells fixed directly in growth medium with 2.6% paraformaldehyde and 50 mM sodium phosphate (pH 7.5), with similar results. For Fig. 3D, overnight cultures were diluted 1:200 into LB0N Amp IPTG (100 μM) either lacking or containing 0.2% l-arabinose and grown at 30°C to OD_600_ = 0.3.

For Fig. 4, overnight cultures were diluted 1:200 into LB or LB0N. Where indicated, LB0N was amended by adding a 10th volume of H_2_O, 2.0 M NaCl, 2.0 M proline, or 2.0 M sucrose. For the strain with the plasmid-based *drpB-gfp* fusion, medium contained both ampicillin (200 μg/ml) and IPTG (25 μM), but these were omitted for the strain with the chromosomal *drpB-gfp* fusion. Samples were taken for Western blotting and microscopy at OD_600_ = 0.5.

For Fig. 5B, overnight cultures were diluted 1:500 in LB and grown at the indicated temperature to OD_600_ = 0.5, then fixed and photographed under phase contrast. For Fig. 5C, overnight cultures were diluted 1:200 into LB and grown at 30°C to OD_600_ = 0.4. At this point cultures were diluted 1:10 into prewarmed LB and grown for 1 h prior to fixing samples and staining membranes with FM4-64 for microscopy.

### Microscopy.

Our system for acquisition of phase contrast and fluorescence micrographs has been described (73).

### Bioinformatic analysis of DrpB.

Homologs of DrpB were identified by Tblastn. Briefly, the nonredundant nucleotide database was queried on 17 March 2020 with E. coli DrpB assuming the residue annotated as M22 is the initiating methionine. Default parameters were as follows: word size = 6; matrix = BLOSUM62; gap costs = 11 (Existence 1); and filtering of low complexity regions. Hits of interest were retrieved and aligned using PSI/TM-Coffee with transmembrane selected and homology search extension set to UniRef100—slow/accurate.

### Bacterial two-hybrid assays.

Transformants of DHM1 (41) carrying appropriate plasmid pairs (derivatives of pUT18 and pKT25) were streaked onto plates of LB medium containing Amp (200 μg/ml) and Kan (40 μg/ml). Plates were incubated for 2 or 3 days at 30°C. Three colonies were used to inoculate 5 ml of LB medium containing Amp, Kan, and 40 μM IPTG. Cultures were grown on a roller at 30°C for 16 h to an OD_600_ of 0.6 to 0.8. Then triplicate 10-μl culture samples were assayed for β-galactosidase activity as described below. Qualitatively similar results were obtained when IPTG was omitted and when strains were assayed visually by spotting onto minimal medium plates containing Amp, Kan, IPTG, and X-gal.

### β-Galactosidase assay procedures.

Overnight cultures were sampled for cell density and β-galactosidase activity as follows: (i) the turbidity was determined by diluting 50 μl of culture with 150 μl of LB in a 96-well flat-bottom plate and measuring the absorbance at 600 nm; (ii) another 120 μl culture was lysed by vigorously pipetting into 6 μl of 2% Sarkosyl and 12 μl chloroform prepared in a 96-well polypropylene PCR plate. After 15 min of settling, 10 μl of the lysate was added to 100 μl of Z-buffer (60 mM Na_2_HPO_4_, 40 mM NaH_2_PO_4_, 10 mM KCl, 1 mM MgSO_4_, 50 mM 2-mercaptoethanol, pH 7.0) in a flat-bottom 96-well plate. The reaction was started by the addition of 50 μl *o*-nitrophenyl-β-d-galactopyranoside (ONPG) (10 μg/ml) and color development was monitored at 405 nm, every 1 min for 30 min. Beta-galactosidase activity was reported as the rate of color development normalized to cell density. All absorbance measurements were performed with a Tecan Infinite M200 Pro plate reader. All assays were repeated at least four times on different days.

## Supplementary Material

Supplemental file 1
