# Supplementary material for: DrpB (YedR) Is a Nonessential Cell Division Protein in Escherichia coli
Source: J Bacteriol. 2020 Nov 4;202(23):e00284-20. doi: 10.1128/JB.00284-20 (PMC7648144; doi:10.1128/JB.00284-20)
Supplement: Supplemental file 1 [file JB.00284-20-s0001.pdf]

Supplemental Material for:

DrpB (YedR) is a non-essential cell division protein in *Escherichia coli*

Atsushi Yahashiri,[a] Jill T. Babor,[a,1] Ariel L. Anwar,[a,2] Ryan P. Bezy,[d] Evan W. Piette,[a,3] S.J. Ryan Arends,[a,4] Ute Müh, [a] Monica R. Steffen,[d,5] Jeremy M. Cline[d,6] David N. Stanek[d,7] Steven D. Lister,[a,8] Shauna M. Swanson [a,9] and and David S. Weiss[a,#]

[a]Department of Microbiology and Immunology, The University of Iowa, Carver College of Medicine, The University of Iowa, Iowa City, IA 52242

[b]Department of Natural and Applied Sciences, Mount Mercy University, Cedar Rapids, IA 52402

Running Head: New *E. coli* cell division protein

#Address correspondence to: David S. Weiss (david-weiss@uiowa.edu)

Present Address

[1]Jill T. Babor, University of Florida, Department of Microbiology and Cell Science, 1355 Museum Dr., Room 1260, Gainesville, FL, 32603

[2]Ariel L. Anwar, Kent State University, Cunningham Hal, 1275 University Esplanade, Kent, OH 44242

[3]Evan W. Piette, M.D., UVM Dermatology, 111 Colchester Ave, Main Campus, East Pavilion, Level 3, Burlington, VT 05401

[4]S.J. Ryan Arends, JMI Laboratories, North Liberty, IA, USA

[5]Monica R. Steffen, 1501 Grand Ave. Ames IA, 50010

[6]Jeremy M. Cline, 4411 Lee St. NE, Cedar Rapids 52402

[7]David N. Stanek, 4615 Windy Meadow Cir NE, Cedar Rapids, IA 52411

[8]Steven D. Lister, 2500 Wiggins Rd, Muscatine, IA 52761

[9]Shauna M. Swanson, Camargo Pharmaceutical Services, Durham, NC 27701

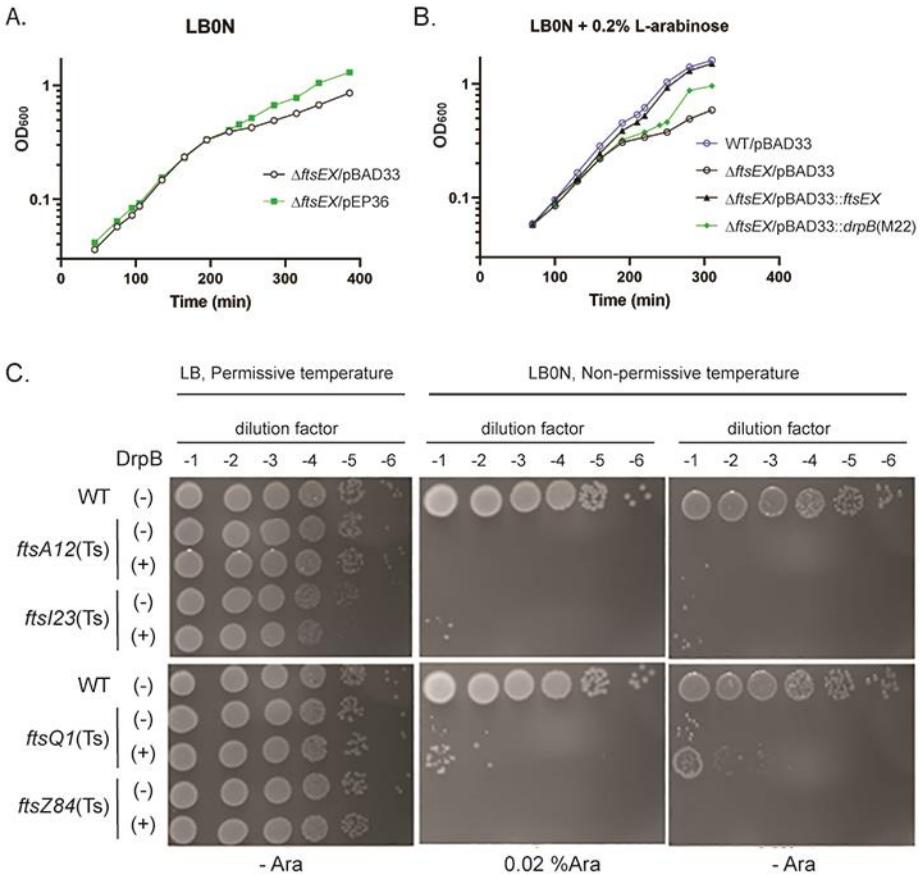

Fig. S1. Effect of overproduction of DrpB on growth of several *fts* mutants. (A, B) Overproduction of DrpB improves growth of the Δ*ftsEX* strain EC1215 in LB0N broth. Overnight cultures grown at 30°C in LB-chloramphenicol were diluted 1:200 into LB0N-chloramphenicol lacking (A) or containing (B) 0.2% arabinose and grown at 30°C with shaking. Growth curves shown are representative of 3 replicates. (C) Overproduction of DrpB fails to rescue several *fts* mutants. Overnight cultures grown in LB-chloramphenicol were adjusted to OD_{600} = 1.0 in LB0N. Ten-fold serial dilutions were prepared in LB0N and 3 µl were spotted onto the indicated plates. Plates were photographed after incubating for 18 h at 30°C or 42°C. All plates contained chloramphenicol. Strains shown are transformants of EC251 (WT), EC295 [*ftsI*(Ts)], EC297 [*ftsA*(Ts)], EC303 [*ftsQ*(Ts)] and EC309 [*ftsZ*(Ts)]. The plasmids are pBAD33 and pDSW1627 [pBAD33::*drpB*].

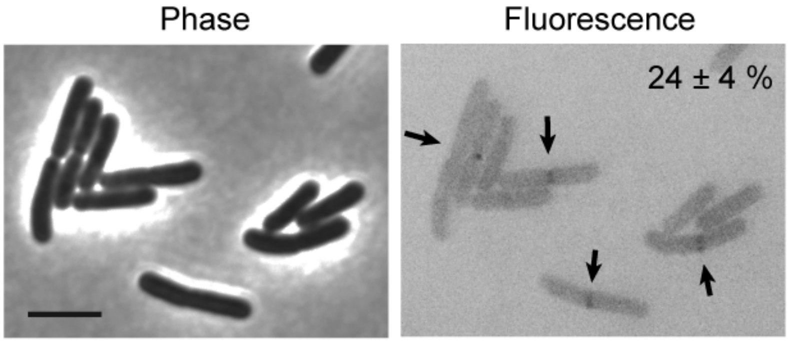

Fig. S2. Localization of DrpB-GFP fusion produced from native chromosomal locus. An overnight culture of EC4680 [*drpB-gfp*, Kan^r] grown at 30°C in LB containing kanamycin (40 μg/ml) was diluted 1:200 into LB0N without any antibiotic and grown at 30°C to OD_{600} = 0.5. Live cells were spotted on an agarose pad and photographed. The fluorescence micrograph was inverted to better visualize DrpB-GFP signal. Arrows point to examples of septal localization. The number in upper right is the percentage of cells exhibiting septal localization (mean ± st. dev. from 3 experiments.)

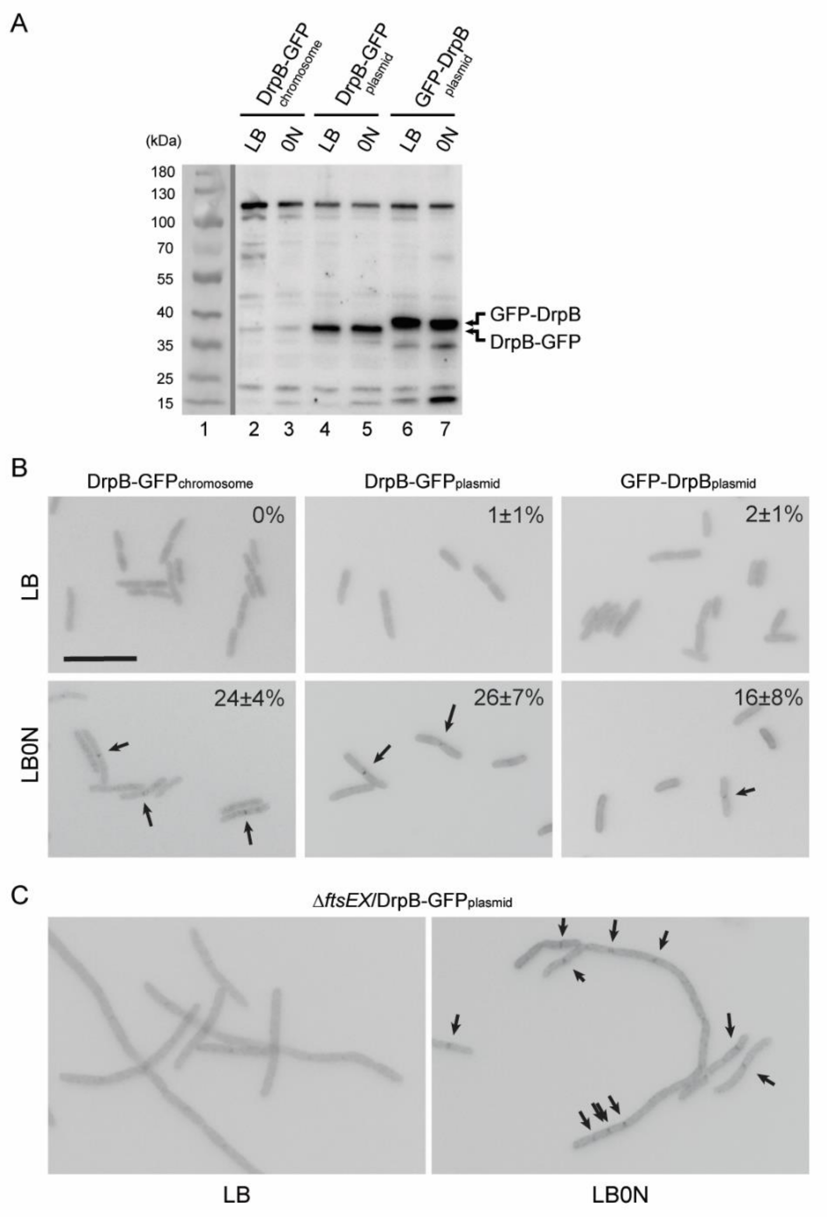

Fig. S3. DrpB localizes to division sites when cells are grown in LB0N but not LB. Starter cultures were grown overnight at 30°C in LB containing appropriate antibiotics then diluted 1:200 into LB or LB0N, supplemented with ampicillin (200 µg/ml) and IPTG (25 µM) for plasmid strains. Cultures were grown at 30°C to OD_{600} = 0.5 before harvesting for Western blotting with anti-GFP antisera (A) or transferring aliquots of live cells onto agarose pads for fluorescence microscopy (B, C). Fluorescence micrographs were inverted to better visualize GFP fusions. Arrows in (B,C) indicate examples of septal localization. Numbers in (B) refer to the percentage of cells exhibiting septal localization (mean ± st. dev. from 2 experiments). Size bar = 10 µm. Strains used were EC4680 (*drpB-gfp* at native chromosomal locus), EC4769 [Δ*drpB*<>*kan*/pDSW1934 (P_{206}::(M22)*drpB-gfp*)] and EC4837 [Δ*drpB*<>*kan*/pDSW1991 (P_{206}::*gfp*-(M22)*drpB*)], and EC4676 [Δ*ftsEX*/pDSW1934 (P_{206}::(M22)*drpB-gfp*].

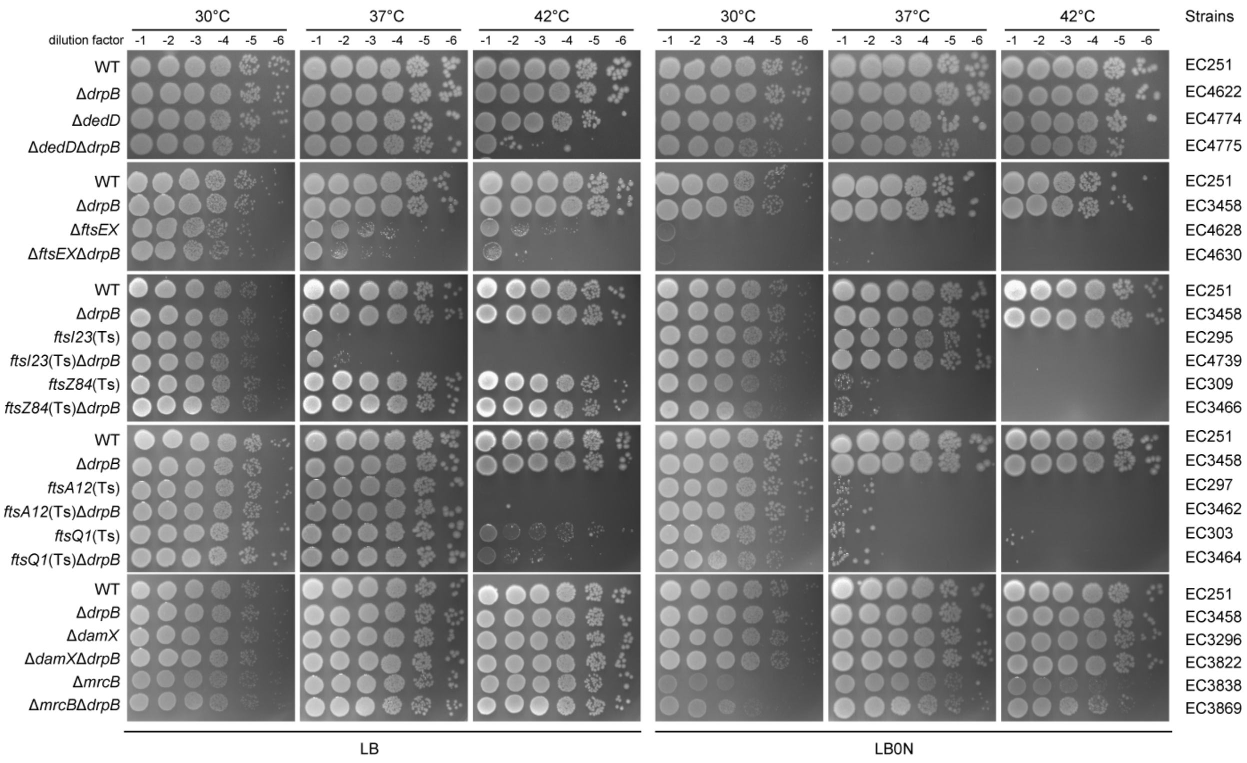

Fig. S4. Plating efficiency of double mutants. Starter cultures were grown overnight at 30°C in LB containing kanamycin or chloramphenicol, if appropriate. Cell densities were adjusted to OD_{600} = 1.0 in LB0N, ten-fold serially diluted in LB0N, and 3 µl were spotted onto LB or LB0N plates. Plates were incubated at the indicated temperatures for 18 h and photographed.

TABLE S1. Effect of pEP36 and pBAD33::*drpB* on cell division[a]

Strain description	Strain number[b]	0.2% arabinose	No. of replicates	Ave length in µm (SD)
WT/pBAD33	EC4511	No	1	6.0 (n.a.)[b]
Δ*ftsEX*/pBAD33	EC5418	No	2	29.4 (3.8)
Δ*ftsEX*/pBAD33	EC5418	Yes	4	27.5 (3.7)
Δ*ftsEX*/pEP36	EC4512	No	3	16.7 (1.9)
Δ*ftsEX*/pBAD33::*ftsEX*	EC5419	Yes	3	8.4 (0.4)
Δ*ftsEX*/pBAD33::*drpB*(M22)	EC5420	No	2	27.4 (2.9)
Δ*ftsEX*/pBAD33::*drpB*(M22)	EC5420	Yes	3	16.6 (0.9)

[a] Cells from cultures grown to OD_{600} =0.45 were photographed under phase contrast. Average lengths were determined for each experimental replicate by measuring at least 100 cells. These averages were used to calculate an overall average and standard deviation.
[b] n.a. = not applicable.

TABLE S2. Strains used in this study

Strain	Relevant genetic marker(s) or features	Source, reference or construction
Cloning and expression hosts		
BL21	*ompT gal dcm hsdS*$_B$(rB$^-$mB$^-$)	Novagen
DY330	W3110 Δ*lacU169 gal-490* λ*c1857* Δ(*cro-bioA*)	(1)
NEB 5-alpha	*fhuA2* Δ(*argF-lacZ)U169 phoA glnV44* ϕ*80*Δ*(lacZ)*M15 *gyrA96*<br>*recA1 relA1 endA1 thi-1 hsdR17*	New England Biolabs
OmniMAX-2 T1R	F′ [*proAB*$^+$ *lacI*^{q} *lacZ*ΔM15 Tn*10*(Tet^R) Δ(*ccdAB*)] *mcrA* Δ(*mrr-hsdRMS-mcrBC*) ϕ80*lacZ*ΔM15<br>Δ(*lacZYA-argF*)*U169 endA1 recA1 supE44 thi-1 gyrA96 relA1 tonA panD*	Invitrogen
PIR2	Δ*lac169 rpoS(Am) robA1 creC510 hsdR514 endA recA1 uidA(*Δ*MluI)::pir*	Invitrogen
XL 1-Blue	F′ [*proAB*$^+$ *lacI*^{q}*lacZ*Δ*M15* Tn*10* (Tet^R)]<br>*recA1 endA1 gyrA96 thi-1 hsdR17 supE44 relA1 lac*	Agilent
Keio strains		
JW0145	BW25113 Δ*mrcB<>kan*	(2)
JW1946	BW25113 Δ*drpB<>kan*	(2)
JW3578	BW25113 Δ*dedD<>kan*	(2)
Weiss lab strains		
EC251	WT, lab isolate of MG1655	(3)
EC295	EC251 *ftsI23*(Ts) *leu*::Tn*10*	(4)
EC297	EC251 *ftsA12*(Ts) *leu*::Tn*10*	(5)
EC303	EC251 *ftsQ1*(Ts)	(6)
EC309	EC251 *ftsZ84*(Ts) *leu*::Tn*10*	(7)
EC1111	EC251 Δ*ftsEX<>cam*<br>*att*$_{HK022}$::pDSW525(P$_{BAD}$-*ftsEX* Spc^R)	(8)
EC1215	EC251 Δ*ftsEX<>frt*	(8)
EC1910	EC251 *damX<>kan*	(6)
EC3296	EC251 Δ*damX<>frt*	Evict *kan* from EC1910 with pCP20
EC3374	EC251/pDSW1642	Transformation
EC3458	EC251 Δ*drpB<>kan*	P1 JW1946 X EC251, select Kan^r
EC3462	EC251 *ftsA12*(Ts) *leu*::Tn*10* Δ*drpB<>kan*	P1 JW1946 X EC297, select Kan^r at 30°C
EC3464	EC251 *ftsQ1*(Ts) Δ*drpB<>kan*	P1 JW1946 X EC303, select Kan^r at 30°C
EC3466	EC251 *ftsZ84*(Ts) *leu*::Tn*10* Δ*drpB<>kan*	P1 JW1946 X EC309, select Kan^r at 30°C
EC3815	EC251 Δ*mrcB<>kan*	P1 JW0145 X EC251, select Kan^r
EC3822	EC251 Δ*damX<>frt* Δ*drpB<>kan*	P1 JW1946 X EC3296, select Kan^r
EC3838	EC251 Δ*mrcB<>frt*	Evict *kan* from EC3815 with pCP20
EC3869	EC251 Δ*mrcB<>frt* Δ*drpB<>kan*	P1 JW1946 X EC3838, select Kan^r
EC4511	EC251/pBAD33	Transformation
EC4512	EC1215/pEP36	Transformation
EC4513	EC1215/pBAD33	Transformation

EC4514	EC1215/pDSW1626	Transformation
EC4515	EC1215/pDSW1627	Transformation
EC4558	EC1215/pDSW1643	Transformation
EC4559	EC1215/pDSW1914	Transformation
EC4560	EC1215/pDSW1916	Transformation
EC4561	EC1215/pDSW1918	Transformation
EC4580	EC1215/pDSW1930	Transformation
EC4581	EC1215/pDSW1931	Transformation
EC4582	EC1215/pDSW1940	Transformation
EC4587	EC251/pDSW1934	Transformation
EC4622	EC251 Δ*drpB<>frt*	Evict *kan* from EC3458 with pCP20
EC4628	EC251 Δ*ftsEX<>cam*	P1 EC1111 X EC251, select Cam^{r} on M9-maltose at 30°C
EC4630	EC251 Δ*ftsEX<>cam* Δ*drpB<>kan*	P1 EC1111 X EC3458, select Cam^{r} on M9-maltose at 30°C
EC4657	DY330 *drpB-gfp kan*	λ Red recombineering to integrate a 2148 bp *'drpB-gfp kan* PCR fragment from pDSW1961 at the *drpB* chromosomal locus of DY330
EC4670	EC251/pDSW210	Transformation
EC4672	EC1215/pDSW210	Transformation
EC4676	EC1215/pDSW1934	Transformation
EC4680	EC251 *drpB-gfp kan*	P1 EC657 X EC251, select Kan^{r}
EC4705	EC297/pBAD33	Transformation
EC4707	EC297/pDSW1627	Transformation
EC4709	EC296/pBAD33	Transformation
EC4711	EC296/pDSW1627	Transformation
EC4713	EC303/pBAD33	Transformation
EC4715	EC303/pDSW1627	Transformation
EC4717	EC309/pBAD33	Transformation
EC4719	EC309/pDSW1627	Transformation
EC4727	EC309/pDSW1642	Transformation
EC4739	EC251 *ftsI23*(Ts) *leu*::Tn*10* Δ*drpB<>kan*	P1 EC295 X EC3458, select Tet^{r} at 30°C.
EC4741	EC1215/pDSW1975	Transformation
EC4742	EC1215/pDSW1977	Transformation
EC4743	EC1215/pDSW1979	Transformation
EC4762	EC4742/pDSW235	Transformation
EC4769	EC251Δ*drpB*<>kan/pDSW1934	Transformation into EC3458
EC4774	EC251 Δ*dedD<>kan*	Transformation
EC4775	EC251 Δ*dedD<>kan* Δ*drpB<>frt*	P1 JW3578 X EC4622, select Kan^{r}
EC4827	EC251 *drpB-gfp frt*	Evict *kan* from EC4680 with pCP20
EC4841	EC251 Δ*dedD<>kan drpB-gfp*	P1 JW3578 X EC4827, select Kan^{r}
EC5418	EC1215/pBAD33	Transformation[a]
EC5419	EC1215/pDSW610	Transformation
EC5420	EC1215/pDSW1977	Transformation[a]

[a]These strains are reconstructions of EC4513 and EC4742. We did this because EC1215 readily acquires suppressor mutations, so we wanted to verify some results with newly constructed strains.

Table S3: Plasmids used in this study.

Plasmid	Relevant Features/description	Source or Reference
pBAD33	Arabinose regulation (P_{BAD}); p15A ori Cm^r	(9)
pCP20	λP_R::*FLP* λcI857 *bla cat* Rep^{TS} (pSC101 derivative)	(10)(10)(10)(10)
pDSW209	P_{206}::*MCS-gfp* Amp^r pBR ori; GFP fusion vector	(11)
pDSW210	P_{206}::*gfp-MCS* Amp^r pBR ori; GFP fusion vector	(11)
pDSW230	P_{204}::*ftsZ-gfp* Amp^r pBR ori	(11)
pDSW235	P_{206}::*gfp-ftsI* (pDSW210 derivative)	(11)
pDSW610	pBAD33::*ftsEX*	(12)
pDSW1626	pBAD33::*rseX* (with 110 bp upstream of transcriptional start site)	This study
pDSW1627	pBAD33::*drpB* (with 75 bp upstream of incorrect GTG start codon)	This study
pDSW1642	pDSW210::*drpB* [P_{206}::*drpB-gfp* Amp^r]	This study
pDSW1643	pBAD33::*drpB**10 (Annotated Ile 10 changed to stop codon)	This study
pDSW1883	pQE80L::*gfp* (to overproduce His_6-GFP)	This study
pDSW1901	P_{206}::*drpB-gfp* (M22 translational start, 64 amino acid linker, pSD286 derivative)	This study
pDSW1914	pBAD33::*drpB**23 (Glu 23 changed to stop codon)	This study
pDSW1916	pBAD33::*drpB**30 (Glu 30 changed to stop codon)	This study
pDSW1918	pBAD33::*drpB**65 (Ser 65 changed to stop codon)	This study
pDSW1930	pBAD33::*drpB* M1A (GTG-1 changed to Ala)	This study
pDSW1931	pBAD33::*drpB* M22A (Met 1 changed to Ala)	This study
pDSW1934	pDSW210::*drpB* M22 (P_{206}::*drpB-gfp* Amp^r; translation of *drpB* initiates at the correct start site, annotated as codon 22)	This study
pDSW1940	pBAD33::*drpB* M29A (Met 22 changed to Ala)	This study
pDSW1955	pBAD33::*drpB* AA (GTG-1 and Met 22 changed to Ala)	This study
pDSW1959	pDSW1642::*frt-kan-frt* [P_{206}::*drpB-gfp frt-kan-frt* Amp^r]	This study
pDSW1961	pJC69::*'drpB-gfp frt-kan-frt*	This study
pDSW1975	pBAD33::*drpB* M1 (Translation can initiate at annotated GTG-1)	This study
pDSW1977	pBAD33::*drpB* M22 (Translation initiates at correct start site, annotate as codon 22)	This study
pDSW1979	pBAD33::*drpB* M29 (Translation initiates at annotated codon 29)	This study
pDSW1991	P_{206}::(M22)*drpB-gfp* (pDSW209 derivative)	This study
pJC69	$oriR_{R6K\gamma}$ $attP_{\phi 80}$ Spc^r (CRIM vector)	(13)
pKD13	$oriR_{R6K\gamma}$ *frt-kan-frt* Amp^r	(14)
pKT25	BACTH vector for fusion of target proteins to *Bordetella pertussis cya* gene T25 fragment; P_{lac}::*cya*$^{1-675}$-MCS p15 ori Kan^R	(15)
pKT25-*damX*	P_{lac}::*cya*$^{1-675}$-*damX*	(6)
pKT25-*dedD*	P_{lac}::*cya*$^{1-675}$-*dedD*	(6)
pKT25-*drpB*	P_{lac}::*cya*$^{1-675}$-*drpB*	This study

pKT25-*blr*	P_{lac}::*cya*$^{1-675}$-*blr*	(16)
pKT25-*ftsA*	P_{lac}::*cya*$^{1-675}$-*ftsA*	(17)
pKT25-*ftsB*	P_{lac}::*cya*$^{1-675}$-*ftsB*	(17)
pKT25-*ftsI*	P_{lac}::*cya*$^{1-675}$-*ftsI*	(17)
pKT25-*ftsL*	P_{lac}::*cya*$^{1-675}$-*ftsL*	(17)
pKT25-*ftsN*	P_{lac}::*cya*$^{1-675}$-*ftsN*	(17)
pKT25-*ftsQ*	P_{lac}::*cya*$^{1-675}$-*ftsQ*	(17)
pKT25-*ftsX*	P_{lac}::*cya*$^{1-675}$-*ftsX*	(17)
pKT25-*ftsZ*	P_{lac}::*cya*$^{1-675}$-*ftsZ*	(17)
pKT25-*malF*	P_{lac}::*cya*$^{1-675}$-*malF*	(17)
pKT25-*malG*	P_{lac}::*cya*$^{1-675}$-*malG*	(17)
pKT25-*pbp2*	P_{lac}::*cya*$^{1-675}$-*pbp2*	This study
pKT25-*ymgF*	P_{lac}::*cya*$^{1-675}$-*ymgF*	(17)
pKNT25-*ymgF*	P_{lac}::*ymgF-cya*$^{1-675}$	(18)
pQE80L	T5 promoter/*lac* operators; *lacI*^{q} Amp^{r} ColE1 ori	QIAGEN
pSD286	P_{204}::*MCS-L60 linker-gfp* Amp^{r} pBR ori	(19)
pUT18	BACTH vector for fusion of target proteins to *B. pertussis cya* gene T18 fragment; P_{lac}::MCS-*cya*$^{675-1197}$ pUC ori Amp^{R}	(15)
pUT18-*drpB*	P_{lac}:: *drpB-cya*$^{675-1197}$ (pDSW1726, pUC ori)	This study
pUT18'-*drpB*	P_{lac}:: *drpB-cya*$^{675-1197}$ (pDSW1863, pBR ori)	This study

pDSW1626: PCR amplify *rseX* with primers P1859 and P1860. The 253 bp product, which includes 110 bp upstream and downstream of the *rseX* small RNA, was digested with SacI and XbaI, then ligated into the same sites of pBAD33.

pDSW1627: PCR amplify *drpB* with primers P1861 and P1862. The 493 bp product, which includes 75 bp upstream of the (mis)annotated GTG start codon, was digested with SacI and XbaI, then ligated into the same sites of pBAD33.

pDSW1642: PCR amplify *drpB* with primers P1875 and P1876. The 387 bp product was digested with EcoRI and PstI, then ligated into the same sites of pDSW210.

pDSW1643: PCR amplify 5' end of *drpB* using primers P1861 andP1877 to generate a 114 bp product. PCR amplify the 3' end of *drpB* using P1878 and P1862 to generate a 422 bp product. The two PCR products were mixed and used as a template to amplify full-length *drpB* with primers P1861 and P1862 to generate a 509 bp product that was digested with SacI and XbaI, then ligated into the same sites of pBAD33.

pDSW1883: PCR amplify *gfp* from pDSW230 using primers P2168 and Trc-rev. The 800 bp product was digested with BamHI and HindIII, then ligated into the same sites of pQE80L.

pDSW1901: PCR amplify *drpB* from pEP36 with primers P2199 and P2200. The 395 bp product was digested with EcoRI and XbaI, then ligated into the same sites of pSD286.

pDSW1914: The NEB Q5 Mutagenesis Kit was used to convert codon 23 to a stop codon (GAA→TAA) with primers P2229 and P2230 and template pDSW1627.

pDSW1916: The NEB Q5 Mutagenesis Kit was used to convert codon 30 to a stop codon (GAA→TAA) with primers P2231 and P2232 and template pDSW1627.

pDSW1918: The NEB Q5 Mutagenesis Kit was used to convert codon 65 to a stop codon (AGC→TGA) with primers P2233 and P2234 and template pDSW1627.

pDSW1930: The NEB Q5 Mutagenesis Kit was used to convert the GTG annotated as codon 1 to GCG (alanine) with primers P2245 and P2246 and template pDSW1627.

pDSW1931: The NEB Q5 Mutagenesis Kit was used to convert the ATG annotated as codon 22 to GCG (alanine) with primers P2247 and P2248 and template pDSW1627.

pDSW1934: PCR amplify *drpB* from ATG (mis)annotated as codon 21 with primers P2241 and P1876. The 303 bp product was digested with EcoRI and PstI, then ligated into the same sites of pDSW210.

pDSW1940: The NEB Q5 Mutagenesis Kit was used to convert the ATG annotated as codon 29 to GCG (alanine) with primers P2249 and P2250 and template pDSW1627.

pDSW1955: The NEB Q5 Mutagenesis Kit was used to convert the ATG annotated as codon 22 to GCG (alanine) with primers P2247 and P2248 and template pDSW1930.

pDSW1959: PCR amplify a 1362 *frt-kan-frt* fragment of pKD13 with primers P2279 and P2274. The PCR product was introduced into HindIII-digested pDSW1642 by Gibson Assembly.

pDSW1961: A 2412 bp EcoRI-KpnI fragment from pDSW1959 was ligated into the same sites of pJC69.

pDSW1975: PCR amplify *drpB* with primers P2237 and P2240. The 399 bp product was digested with KpnI and HindIII, then ligated into the same sites of pBAD33. The forward primer P2237 furnishes a Shine-Dalgarno and changes the GTG (mis)annotated as codon 1 to an ATG.

pDSW1977: PCR amplify *drpB* with primers P2238 and P2240. The 316 bp product was digested with NdeI and HindIII, then ligated into the same sites of pBAD33. The forward primer P2238 furnishes a Shine-Dalgarno coupled to the *drpB* ATG (mis)annotated as codon 22.

pDSW1979: PCR amplify *drpB* with primers P2239 and P2240. The 298 bp product was digested with NdeI and HindIII, then ligated into the same sites of pBAD33. The forward primer P2239 furnishes a Shine-Dalgarno coupled to the *drpB* ATG (mis)annotated as codon 29.

pDSW1991: PCR amplify *drpB* from pDSW1627 with primers P2331 and P2332. The 334 bp product was digested with EcoRI and HindIII, then ligated into the same sites of pDSW209.

pUT18-*drpB*: Also known as pDSW1726. PCR amplify *drpB* with primers P1897 and P1899. The 387 bp product was digested with BamHI and KpnI, then ligated into the same sites of pUT18.

pUT18'-*drpB*: Also known as pDSW1863. The 1257 bp PvuI-PciI restriction fragment from pUT18 was replaced with the corresponding fragment from pDSW207, resulting in exchange of a high copy pUC ori for a medium copy pBR ori.

pKT25-*drpB*: Amplify *drpB* with P1897 and P1898. The 387 bp product was cut with BamHI and KpnI, then ligated into same sites of pKT25. Also known as pDSW1723.

pKT25-*pbp2*: Amplify *mrdA* (encodes PBP2) with primers P2174 and P2175. The 1953 bp product was inserted into BamHI-EcoRI digested pKT25 by Gibson Assembly.

Table S4. Oligonucleotides used in this study

Name	Sequence (5'->3')	Comment
Trc-rev	GTTCTGATTTAATCTGTATCAGGC	Reverse primer to clone *gfp* into pQE80L
P959	AACGACAGGAGCACGATCATGCG	Sequencing primer for pBR library
P960	CCTGACGTCTAAGAAACCATTATTATC	Sequencing primer for pBR library
P971	CAAATGTAGCACCTGAAGTCAGCC	Sequencing primer for p15A library
P972	GCACGATCATGCGCACCCGTG	Sequencing primer for p15A library
P1119	GCCGCAATGGCGGACGCTAAA	Confirm Δ*dedD<>kan* by colony PCR
P1120	AATGCGTGTCGGATGCGGCGTA	Confirm Δ*dedD<>kan* by colony PCR
P1195	TCAGCGTAACAACAAGAAAGGTC	Confirm Δ*damX<>kan* and Δ*damX<>frt* by colony PCR
P1196	TAACTAATTACACCTTCTCCGGCTG	Confirm Δ*damX<>kan* and Δ*damX<>frt* by colony PCR
P1990	GCGCGCACGTTCACCCTCACCC	Confirm Δ*mrcB<>kan* and Δ*mrcB<>frt* by colony PCR
P1991	CTGAAGGGTTAATAAACAACCAGATG	Confirm Δ*mrcB<>kan* and Δ*mrcB<>frt* by colony PCR
P1994	CGGAAGCATCATGACACAGAATA	Confirm Δ*drpB<>kan* and Δ*drpB<>frt* and *drp-gfp* by colony PCR
P1995	GTCCTGGTGCTGATGATGG	Confirm Δ*drpB<>kan* and Δ*drpB<>frt* and *drp-gfp* by colony PCR
P1859	CCAGAGCTCCGCAACGGCAGTGAATACAG	Forward primer for cloning *rseX* into pBAD33
P1860	GCGTCTAGAATTGTTGCGCCAAACGGCTG	Reverse primer for cloning *rseX* into pBAD33
P1861	GCGGAGCTCCCTGTATTCACTGCCGTTGC	Forward primer for cloning *drpB* into pBAD33
P1862	GCGTCTAGATGTAAGTCCATACGCGCTCC	Reverse primer for cloning *drpB* into pBAD33
P1875	CACGAATTCGAAAAATGTGACTTTTATCAC	Forward primer for cloning *drpB* into pDSW210 to construct *drpB-gfp* fusion
P1876	CACCTGCAGGTTGTTGTTTTCATAGCGTCTGCTACGTGC	Reverse primer for cloning *drpB* into pDSW210 to construct *drpB-gfp* fusion

P1877	TCATATGTGATAAAAGTCACATTTTTCC	Mutagenizing Ile10 to stop codon
P1878	GAAAAATGTGACTTTTATCACATATGAGTACTAAGTCTGAATTTTCCGG	Mutagenizing Ile10 to stop codon
P1897	CTAGAGGATCCCGTGGAAAAATGTGACTTTTATCAC	Forward primer for cloning *drpB* into pUT18 and pKT25
P1898	CTTAGGTACCTTATTCATAGCGTCTGCTACGTGC	Reverse primer for cloning *drpB* into pKT25
P1899	CTTAGGTACCAATTCATAGCGTCTGCTACGTGC	Reverse primer for cloning *drpB* into pUT18
P2168	GCTGATAACGGATCCAACAACAACATGAGTAAAGGAGAAGAACTTTTCA CTGGAGTTGTC	Forward primer for cloning *gfp* into pQE80L
P2174	ctgcagggtcgactctagaggatcccATGAAACTACAGAACTCTTTTCGC	Forward primer for cloning *mrdA* into pKT25
P2175	ttgtaaaacgacggccagtgaattcTTAATGGTCCTCCGCTGC	Reverse primer for cloning *mrdA* into pKT25
P2199	CACGAATTCTAAGGACATCCAATATGGAAAAATGTGAC	Forward primer for cloning *drpB* into pSD286
P2200	CACTCTAGATTCATAGCGTCTGCTACGTG	Reverse primer for cloning *drpB* into pSD286
P2229	TCTCAAAATGTAATACGGTTCG	Mutagenizing Glu23 to stop codon
P2230	TAACCCGGAAAATTCAGAC	Mutagenizing Glu23 to stop codon
P2231	GACAAAGATGTAAGAGAGACTCTC	Mutagenizing Glu30 to stop codon
P2232	GAACCGTATTCCATTTTG	Mutagenizing Glu30 to stop codon
P2233	CTGGGTGATGTGACGGATTCCCG	Mutagenizing Ser65 to stop codon
P2234	ATGTAACGCGCCATTGCC	Mutagenizing Ser65 to stop codon
P2237	CGCGGTACCTAAGAAGGAGATATACATGTGGAAAAATGTGAC	Forward primer to clone (M1)*drpB* into pBAD33
P2238	GGCGCATATGGAATACGGTTCGACAAAGATGG	Forward primer to clone (M22)*drpB* into pBAD33
P2239	GGCGCATATGGAATACGGTTCGACAAAGATGG	Forward primer to clone (M29)*drpB* into pBAD33
P2240	CGAAGCTTATTCATAGCGTCTGCTACGTGC	Reverse primer for P2237, P2238 and P2239.
P2241	GCGGAATTCGGTTCGACAAAGATGGAAG	Forward primer to clone (M22)*drpB* into pDSW210
P2245	TCATCCAATGCGGAAAAATGTG	Convert GTG-1 to Ala codon
P2246	GCCAGTCAAGACTCAATC	Convert GTG-1 to Ala codon
P2247	TTATCTCAAAGCGGAATACGGTTCGAC	Convert ATG-22 to Ala codon

P2248	CCCGGAAAATTCAGACTTAG	Convert ATG-22 to Ala codon
P2249	TTCGACAAAGGCGGAAGAGAGAC	Convert ATG-29 to Ala codon
P2250	CCGTATTCCATTTTGAGATAAC	Convert ATG-29 to Ala codon
P2274	ctcatccgccaaaacagccaagcttggtaccGTGTAGGCTGGAGCTGCTTC	Amplify *frt-kan-frt* cassette from pKD13
P2279	ATGGATGAACTATACAAATAACTCGAGATTCCGGGGATCCGTCGAC	Amplify *frt-kan-frt* cassette from pKD13
P2283	TGCCAGCGGTGACGATATTC	Confirm Δ*ftsEX<>frt* and Δ*ftsEX<>cam* by colony PCR
P2284	ACTTTCTCCACGCCTTGCTC	Confirm Δ*ftsEX<>frt* and Δ*ftsEX<>cam* by colony PCR
P2331	CACGAATTCAATAATAATtctggaATGGAATACGGTTCGACAAAG	Forward primer to clone (M22)*drpB* into pDSW209
P2332	CACAAGCTTATTCATAGCGTCTGCTACG	Reverse primer to clone (M22)*drpB* into pDSW209

SUPPLEMENTAL REFERENCES

1. Yu D, Ellis HM, Lee EC, Jenkins NA, Copeland NG, Court DL. 2000. An efficient recombination system for chromosome engineering in *Escherichia coli*. Proc Natl Acad Sci U S A 97:5978-83.
2. Baba T, Ara T, Hasegawa M, Takai Y, Okumura Y, Baba M, Datsenko KA, Tomita M, Wanner BL, Mori H. 2006. Construction of *Escherichia coli* K-12 in-frame, single-gene knockout mutants: the Keio collection. Mol Syst Biol 2:2006 0008.
3. Arends SJ, Weiss DS. 2004. Inhibiting cell division in *Escherichia coli* has little if any effect on gene expression. J Bacteriol 186:880-4.
4. Wissel MC, Weiss DS. 2004. Genetic analysis of the cell division protein FtsI (PBP3): amino acid substitutions that impair septal localization of FtsI and recruitment of FtsN. J Bacteriol 186:490-502.
5. Eberhardt C, Kuerschner L, Weiss DS. 2003. Probing the catalytic activity of a cell division-specific transpeptidase in vivo with beta-lactams. J Bacteriol 185:3726-34.
6. Arends SJ, Williams K, Scott RJ, Rolong S, Popham DL, Weiss DS. 2010. Discovery and characterization of three new *Escherichia coli* septal ring proteins that contain a SPOR domain: DamX, DedD, and RlpA. J Bacteriol 192:242-55.
7. Tarry M, Arends SJ, Roversi P, Piette E, Sargent F, Berks BC, Weiss DS, Lea SM. 2009. The *Escherichia coli* cell division protein and model Tat substrate SufI (FtsP) localizes to the septal ring and has a multicopper oxidase-like structure. J Mol Biol 386:504-19.
8. Arends SJ, Kustusch RJ, Weiss DS. 2009. ATP-binding site lesions in FtsE impair cell division. J Bacteriol 191:3772-84.

9. Guzman LM, Belin D, Carson MJ, Beckwith J. 1995. Tight regulation, modulation, and high-level expression by vectors containing the arabinose P_{BAD} promoter. J Bacteriol 177:4121-30.

10. Cherepanov PP, Wackernagel W. 1995. Gene disruption in *Escherichia coli*: TcR and KmR cassettes with the option of Flp-catalyzed excision of the antibiotic-resistance determinant. Gene 158:9-14.

11. Weiss DS, Chen JC, Ghigo JM, Boyd D, Beckwith J. 1999. Localization of FtsI (PBP3) to the septal ring requires its membrane anchor, the Z ring, FtsA, FtsQ, and FtsL. J Bacteriol 181:508-20.

12. Schmidt KL, Peterson ND, Kustusch RJ, Wissel MC, Graham B, Phillips GJ, Weiss DS. 2004. A predicted ABC transporter, FtsEX, is needed for cell division in *Escherichia coli*. J Bacteriol 186:785-93.

13. Chen JC, Beckwith J. 2001. FtsQ, FtsL and FtsI require FtsK, but not FtsN, for co-localization with FtsZ during *Escherichia coli* cell division. Mol Microbiol 42:395-413.

14. Datsenko KA, Wanner BL. 2000. One-step inactivation of chromosomal genes in *Escherichia coli* K-12 using PCR products. Proc Natl Acad Sci U S A 97:6640-5.

15. Karimova G, Ullmann A, Ladant D. 2001. Protein-protein interaction between *Bacillus stearothermophilus* tyrosyl-tRNA synthetase subdomains revealed by a bacterial two-hybrid system. J Mol Microbiol Biotechnol 3:73-82.

16. Karimova G, Davi M, Ladant D. 2012. The beta-lactam resistance protein Blr, a small membrane polypeptide, is a component of the *Escherichia coli* cell division machinery. J Bacteriol 194:5576-88.

17. Karimova G, Dautin N, Ladant D. 2005. Interaction network among *Escherichia coli* membrane proteins involved in cell division as revealed by bacterial two-hybrid analysis. J Bacteriol 187:2233-43.

18. Karimova G, Robichon C, Ladant D. 2009. Characterization of YmgF, a 72-residue inner membrane protein that associates with the *Escherichia coli* cell division machinery. J Bacteriol 191:333-46.

19. Du S, Lutkenhaus J. 2017. The N-succinyl-l,l-diaminopimelic acid desuccinylase DapE acts through ZapB to promote septum formation in *Escherichia coli*. Mol Microbiol 105:326-345.
